# Transcriptome analysis of seed dormancy after rinsing and chilling in ornamental peaches (*Prunus persica* (L.) Batsch)

**DOI:** 10.1186/s12864-016-2973-y

**Published:** 2016-08-08

**Authors:** Worarad Kanjana, Tomohiro Suzuki, Kazuo Ishii, Toshinori Kozaki, Masayuki Iigo, Kenji Yamane

**Affiliations:** 1 United Graduate School of Agricultural Science, Tokyo University of Agriculture and Technology, Fuchu, Tokyo 183-8509 Japan; 2 Bioscience Education and Research Center, Utsunomiya University, Utsunomiya, Tochigi 321-8505 Japan; 3 Department of Applied Biological Science, Faculty of Agriculture, Tokyo University of Agriculture and Technology, Fuchu, Tokyo 183-8509 Japan; 4 Faculty of Agriculture, Utsunomiya University, Utsunomiya, Tochigi 321-8505 Japan

**Keywords:** Transcriptome, Seed germination, Rinsing, Chilling, Ornamental peach

## Abstract

**Background:**

Ornamental peaches cv. ‘Yaguchi’ (*Prunus persica* (L.) Batsch) can be propagated via seeds. The establishment of efficient seed treatments for early germination and seedling growth is required to shorten nursery and breeding periods. It is important, therefore, to identify potential candidate genes responsible for the effects of rinsing and chilling on seed germination. We hypothesized that longer rinsing combined with chilling of seeds can alter the genes expression in related to dormancy and then raise the germination rate in the peach. To date, most molecular studies in peaches have involved structural genomics, and few transcriptome studies of seed germination have been conducted. In this study, we investigated the function of key seed dormancy-related genes using next-generation sequencing to profile the transcriptomes involved in seed dormancy in peaches. *De novo* assembly and analysis of the transcriptome identified differentially expressed and unique genes present in this fruit.

**Results:**

*De novo* RNA-sequencing of peach was performed using the Illumina Miseq 2000 system. Paired-end sequence from mRNAs generated high quality sequence reads (9,049,964, 10,026,362 and 10,101,918 reads) from ‘Yaguchi’ peach seeds before rinsed (BR) and after rinsed for 2 or 7 days with a chilling period of 4 weeks (termed 2D4W and 7D4W), respectively. The germination rate of 7D4W was significantly higher than that of 2D4W. In total, we obtained 51,366 unique sequences. Differential expression analysis identified 7752, 8469 and 506 differentially expressed genes from BR *vs* 2D4W, BR *vs* 7D4W and 2D4W *vs* 7D4W libraries respectively, filtered based on *p-value* and an adjusted false discovery rate of less than 0.05. This study identified genes associated with the rinsing and chilling process that included those associated with phytohormones, the stress response and transcription factors. 7D4W treatment downregulated genes involved in ABA synthesis, catabolism and signaling pathways, which eventually suppressed abscisic acid activity and consequently promoted germination and seedling growth. Stress response genes were also downregulated by the 7D4W treatment, suggesting that this treatment released seeds from endodormancy. Transcription factors were upregulated by the BR and 2D4W treatment, suggesting that they play important roles in maintaining seed dormancy.

**Conclusions:**

This work indicated that longer rinsing combined with chilling affects gene expression and germination rate, and identified potential candidate genes responsible for dormancy progression in seeds of ‘Yaguchi’ peach. The results could be used to develop breeding programs and will aid future functional genomic research in peaches and other fruit trees.

**Electronic supplementary material:**

The online version of this article (doi:10.1186/s12864-016-2973-y) contains supplementary material, which is available to authorized users.

## Background

Peach (*Prunus persica* (L.) Batsch) is a deciduous tree of the rose family (*Rosaceae*). Peaches are not only widely planted as fruit trees, but also as ornamental plants such as garden trees and cut branches. Budding and grafting are common asexual propagation techniques for fruit trees except breeding purpose because fruit phenotype cannot be maintained via seed propagation. However, seed propagation is possible for ornamental peaches such as ‘Yaguchi’ and ‘Hokimomo’ because they have been repeatedly propagated via seed for a long time and thus genetically homogeneous. Early germination is required to shorten nursery and breeding periods.

Seed germination depends on a number of factors, including internal dormancy and the environment [1]. Seed dormancy is observed in higher plants with distinct physiological and morphological characteristics in different species [2]; however, it is an undesirable characteristic in an agricultural crop where rapid germination and growth are required. Moist chilling plays an important role in providing the stimulus required to overcome dormancy, increase germination and produce normal seedlings in ‘GF305’ peaches [3], strawberry trees [4] and sweet cherries (*Prunus avium)* [5]. Seeds of most *Prunus* species require a period of chilling to break seed dormancy [6–9]. In peaches, removal of the seed coat shortens the chilling periods needed to break dormancy and even improves the germination of non-chilled seeds [10, 11]. When the cold treatment is insufficient, seedlings show physiological dwarfing, which is considered a special case of embryo dormancy [12, 13]. These results implied that dormancy in peach seeds is caused by exogenous and endogenous dormancy associated with the seed covering layers and the embryo [14].

The ratio of the hormones abscisic acid (ABA) and gibberellic acid (GA) is considered a relevant factor regulating seed dormancy. Moist chilling induced an increase in GA levels in embryos of the European hazel (*Corylus avellana*), suggesting that GAs synthesized during cold treatment were responsible for breaking dormancy [15]. ABA plays a key role in various aspects of plant growth. In the ABA biosynthetic pathway, 9-*cis*-epoxycarotenoid dioxygenase (NCED) is the key enzyme in ABA biosynthesis in higher plants [16]. The pattern of *PacNCED1* expression was coincident with that of ABA accumulation in sweet cherry fruit [17]. Recent studies indicated that the key step of ABA inactivation is the hydroxylation of the 8′-methyl group of ABA in most plant tissues. ABA 8′-Hydroxylase is a key enzyme in the oxidative catabolism of ABA and is expressed throughout sweet cherry fruit development.

Germination commences with the uptake of water by imbibition by the dry seed, followed by embryo expansion. In our previous study [18], seeds after rinsing with running tap water for 2 days and chilling at 5 °C for more than 8 weeks showed decreased ABA contents in the embryonic axis and seed coat, which ultimately increased the uniform germination and normal growth in ‘Yaguchi’ peaches. Moreover, longer rinsing (about 8 days) increased germination rate and significantly increased the plant height in ‘Hokimomo’ peach [19]. In *Vitis vinifera*, rinsing with running water for 8 days increased germination rate from 14 to 34 % compared to 4 days [20].

Therefore, we hypothesized that longer rinsing of seeds can alter the genes expression in related to dormancy and then raise the germination rate in ‘Yaguchi’ peach. In the present study, germination rate of seeds after rinsed for 2 days (2D) and 7 days (7D) and chilled for 4 weeks each (termed 2D4W and 7D4W, respectively) were determined. The aim of the present study was to perform transcriptome analysis among seeds of BR, 2D4W and 7D4W seeds and to gain an understanding of molecular mechanism during peach seed dormancy and to prove the hypothesis.

In recent years, RNA-sequencing (RNA-Seq) technology has become the most popular and powerful tool for transcriptome analysis. RNA-Seq is cheaper, more efficient, more sensitive and more accurate in generating transcriptome profiles than microarray analysis and other techniques [21–28]. We use RNA-Seq technology to identify and characterize the expression of the large number of genes, especially those differentially expressed during dormancy progression.

## Results

### The germination rate of embryo in peach

**Fig. 1:**
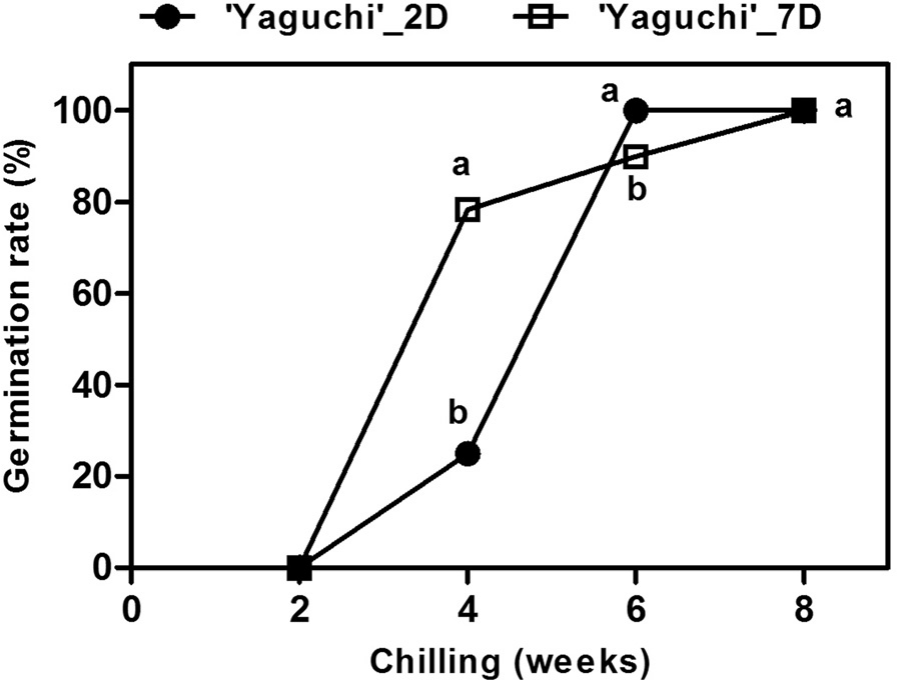
Effects of rinsing and chilling treatments on seed germination. The horizontal axis is peach seeds after distinct periods of chilling after rinsing for 2 and 7 days (2D and 7D). Non-overlapping letters (a–b) indicate significant difference between treatments, based on ANOVA analysis and Multiple Range Tests procedure with a confidence level of 95 %

The germination rate of ‘Yaguchi’ after rinsing for 7 days was significantly higher than that after rinsing for 2 days when the chilling period was 4 weeks (Fig. 1). Chilling periods of between 6 and 8 weeks promoted seed dormancy breaking and increased the final germination rate to 90–100 % (Fig. 1) and resulted in heights of around 15–20 cm, while rinsing for 7 days significantly increased the height, even after 4 weeks of chilling (see Additional file 1). These data the seedling initial germinate after 2D4W and 7D4W each. Therefore, we analyzed the transcriptome only in dry seed before rinsing (BR), 2D4W and 7D4W in ‘Yaguchi’ peaches using next-generation sequencing.

### *De novo* transcriptome assembly and assessments of expressed sequenced tags

#### Sequencing and de novo assembly

Paired-end (PE) sequences from mRNAs generated 9,286,402 (4,643,201 pairs), 10,275,700 reads (5,137,850 pairs) and 10,334,536 reads (5,167,268 pairs) from BR, 2D4W and 7D4W, respectively (Table 1). The regions with low quality scores in the fastq files (quality scores < 30) and reads containing one or more indistinct nucleotides in the raw sequencing data were removed. High quality transcriptome sequence reads (9,049,964, 10,026,362 and 10,101,918 reads) were preprocessed for further analysis (Table 1). To further evaluate the quality of the assemblies, the high quality reads were mapped back to the constructed assemblies (see additional file 2). Almost 95 % of the reads were successfully mapped back to their assemblies. We have also mapped our reads to the total transcript *Prunus persica* annotation v2.1 on assembly v2.0 [29]. BR, 2D4W and 7D4W showed the mapping percentages of 75.85, 74.41 and 75.17 %, respectively. A large number of reads were well matched with peach genes. This could show high accuracy of our contigs assembly. However, the mapping of *de novo* assemblies was higher than *Prunus persica* all transcripts mapped, therefore, in this study we used data from *de novo* transcriptome assemblies for the future analysis. The high-quality short reads from transcriptome sequence reads were assembled into unigenes using the Trinity program. rRNA sequences were excluded from unigenes by removing those matched to the SILVA rRNA database. Ultimately, we obtained 51,366 unique sequences with an average sequence length of approximately 1013 bp; the total length of the sequence was 52,067,809 bases (Table 2). N50 is a statistic widely used to assess the quality of sequence assembly. High values of N50 in our data can indicate the effectiveness of assemblies (Table 2). The variation in N50 may be due to differences between tissues and/or treatments, which can be observed in the previous studies [30, 31].

**Table 1:** The numbers of reads

sample	Raw NGS data		Quality filtered data	
	Reads	Mean quality value	Reads	Mean quality value
Transcriptome of BR (PE reads)	9,286,402	36.93	9,049,964	37.17
Transcriptome of 2D4W (PE reads)	10,275,700	33.31	10,026,362	36.97
Transcriptome of 7D4W (PE reads)	10,334,536	36.68	10,101,918	36.90

**Table 2:** The summary of assembly

Total sequence	51,366
Total bases	52,067,809
Min sequence length (bp)	224
Max sequence length (bp)	10,795
Average sequence length (bp)	1013
N50 length (bp)	1628
(G + C)s	42.55 %

### Differentially expressed genes analysis (DEGs)

We compared the gene expressions among BR, 2D4W and 7D4W data and identified differentially expressed genes. Normalization was applied to the treatments to provide accurate differential expression rather than individual quantification. Total DEGs patterns for each sample are presented in Additional file 3. The significantly DEGs with FDRs < 0.05 obtained by comparing BR *vs* 2D4W, BR *vs* 7D4W and 2D4W *vs* 7D4W libraries were 7752, 8469 and 506 respectively (see Additional file 4). The distributions of DEGs were further analyzed using edgeR package. DEGs are visualized as an MA plot (log ratio versus abundance plot) for set of experiments (BR *vs* 2D4W, BR *vs* 7D4W and 2D4W *vs* 7D4W, see Additional file 5). The red dots highlight transcripts of positive and negative values of log2 Fold Change (logFC), indicating that the sequences were upregulated and downregulated genes in each treatment. The genes names, fold changes, and *p* values for up- and down-regulated DEGs in each treatment were listed in Additional file 4.

### Functional annotation of DEGs

For annotation, the consensus sequences were first searched against the Swiss Institute of Bioinformatics databases (SwissProt) database [32] by local BLASTX (*E*-value cut-off was set at 1e-5) to search for the maximum number of similar genes. Among the 7752 DEGs in BR *vs* 2D4W, 3121 and 4631 were up- and down-regulated genes. From 8469 DEGs of BR *vs* 7D4W, 3290 and 5179 were up- and down-regulated. 377 and 129 DEGs were up- and down-regulated genes in 2D4W *vs* 7D4W (see Additional file 4). These genes were identified from BLAST nr database, SwissProt and Uniprot database and assigned with Kyoto Encyclopedia of Genes and Genomes (KEGG) [33] and Gene ontology (GO) terms in the biological process, molecular functions and cellular component categories [34].

### Functional classification by KEGG

KEGG pathway database records networks of molecular interactions in cells, and variants of them, specific to particular organisms. Pathway-based analysis helps to understand the biological functions of gene products. Pathway information for all annotated sequences was obtained from KEGG pathway annotations. In total, 7752 DEGs of BR *vs* 2D4W, 8469 DEGs of BR *vs* 7D4W and 506 DEGs in 2D4W *vs* 7D4W, 2789, 2982 and 142 sequences, respectively were mapped to the reference pathway in KEGG (see Additional file 4). We focused our attention on carotenoid biosynthesis (see Additional file 6). In the pathways of carotenoid biosynthesis and plant hormone transduction, NCED (EC: 1.13.11.51), ABA 8′-hydroxylase (EC: 1.14.13.93), abscisic acid receptor PYR/PYL family (PYR/PYL), serine/threonine-protein kinase SRK2 (SNRK2), protein phosphatase 2C (PP2C) and ABA responsive element binding factor (ABF) were known to have important roles in the seed dormancy and germination [35].

### GO enrichment analysis of DEGs

**Fig. 2:**
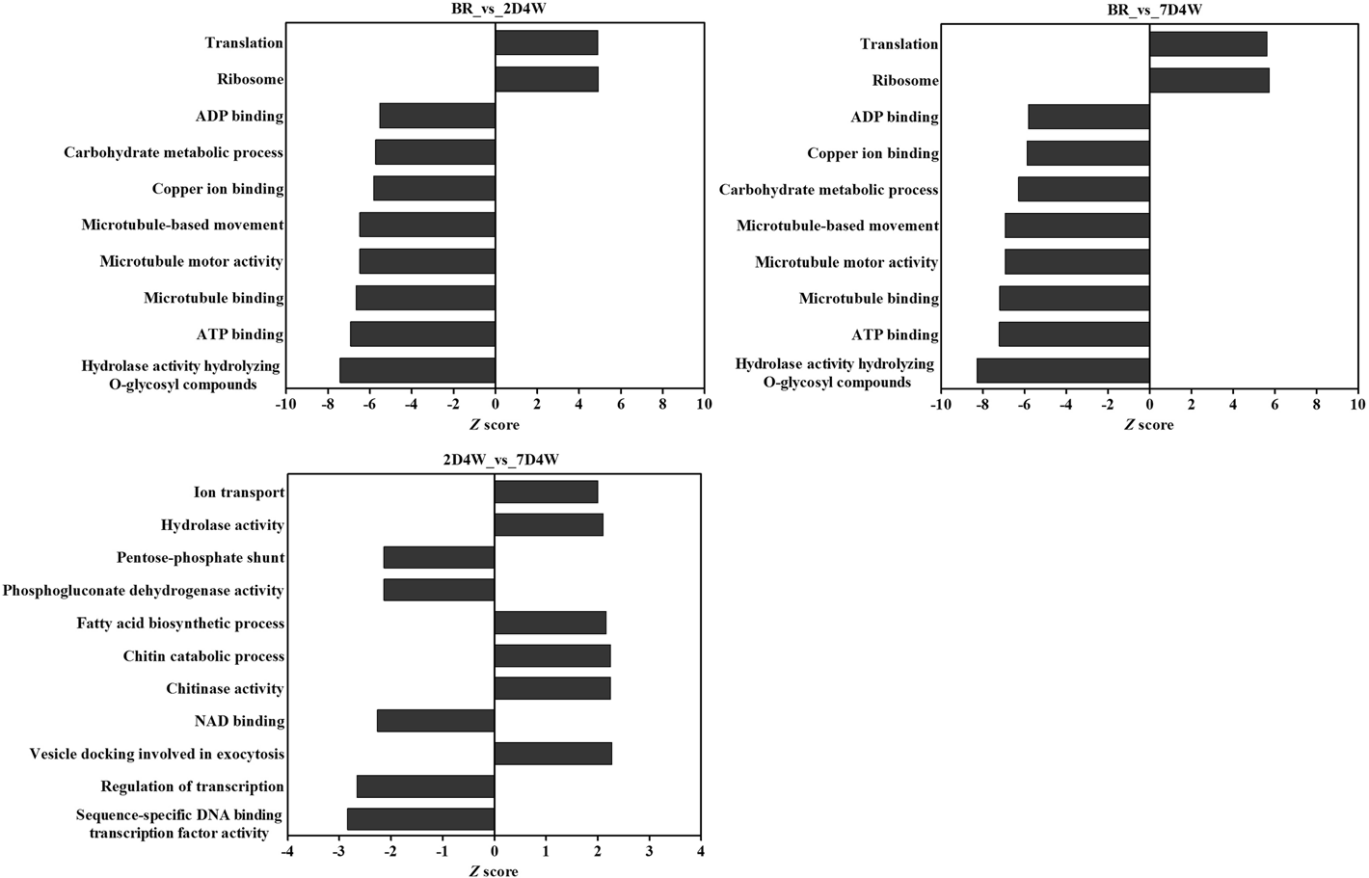
GO enrichment analysis. Enrichment of gene ontology terms in differentially expressed sequences in BR *vs* 2D4W, BR *vs* 7D4W and 2D4W *vs* 7D4W

When significant DEGs were subjected to GO enrichment analysis, most of them were significantly represented in the three main GO categories of ‘biological process’, ‘molecular function’ and ‘cell component’. In the top hit GO molecular function categories, hydrolase activity, hydrolyzing O-glycosyl compounds, ATP binding and microtubule binding were significantly enriched between BR *vs* 2D4W and were also highly enriched in the BR *vs* 7D4W comparison. Sequence-specific DNA binding transcription factor activity, NAD binding and chitinase activity were the most highly enriched terms in 2D4W* vs*. 7D4W. In the GO biological process, microtubule-based movement, carbohydrate metabolic process and translation were significantly enriched between BR *vs* 2D4W and BR *vs* 7D4W, while, regulation of transcription, DNA-templated, vesicle docking involved in exocytosis and chitin catabolic process were the most highly enriched terms in 2D4W *vs*. 7D4W. Moreover, ribosomes were found to be significantly enriched in term of cellular components (see Additional file 7; Fig. 2).

### Quantitative real-time reverse transcription PCR (qRT-PCR) validation of DEGs from transcriptome analysis

**Fig. 3:**
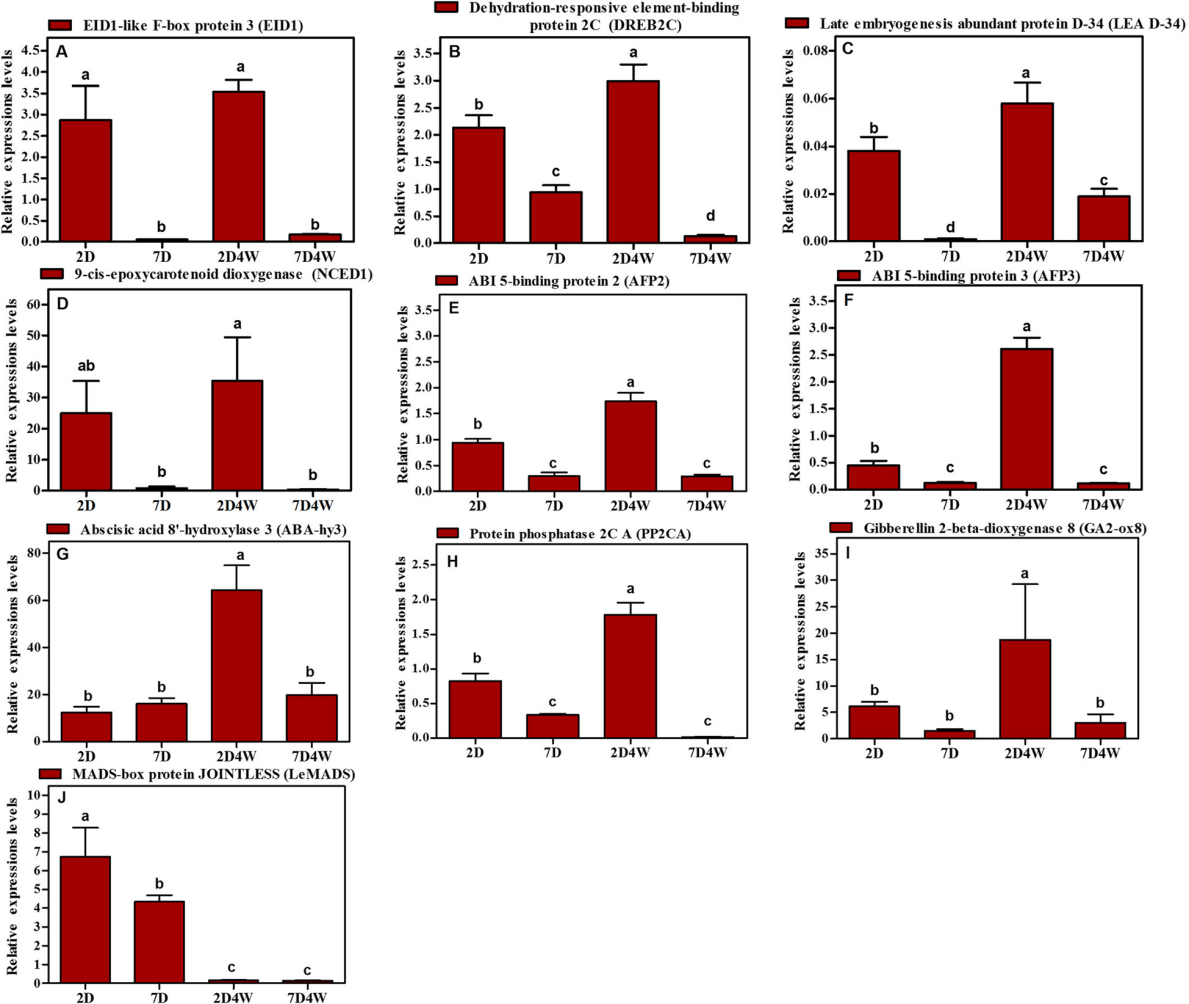
Verification of 10-gene expression patterns by qRT-PCR. Vertical axis indicates relative gene expression levels and horizontal axis is seed peach treatment. Error bars depict the standard error of the mean for three biological replicates. Non-overlapping letters (*a*–*d*) indicate significant difference between treatments, based on ANOVA analysis and Multiple Range Tests procedure with a confidence level of 95 %

**Fig. 4:**
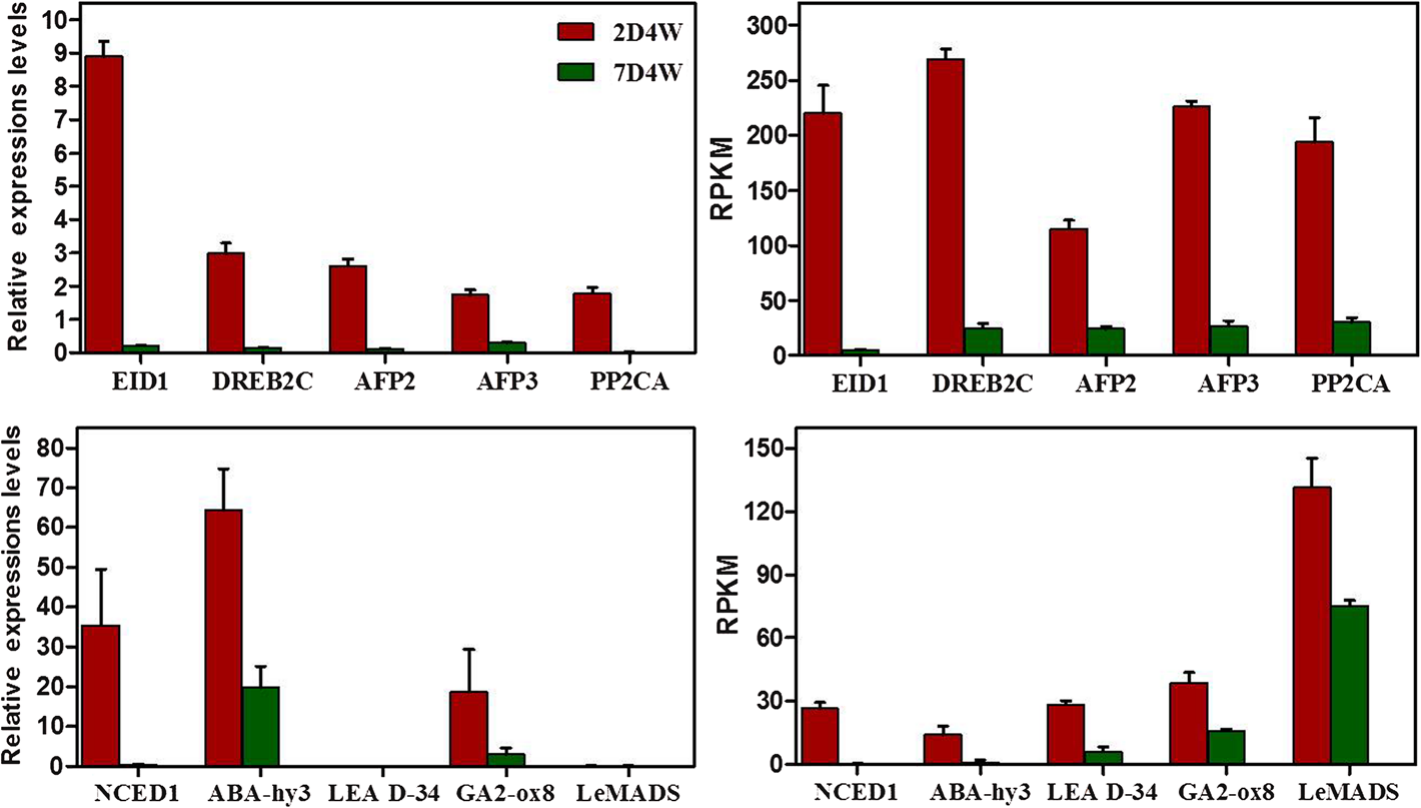
qRT-PCR validation of differential gene expression. The left side indicates relative gene expression levels determined by qRT-PCR after rinsing 2, 7 days and chilling 4 weeks. The right side indicates RPKM levels calculated by the RSEM method. Error bars depict the standard error of the mean for three biological replicates. Coefficient analysis between gene expression ratios obtained from qRT-PCR and RNA-seq data. The correlation coefficient r values of the EID1, DREB2C, AFP2, AFP3, PP2CA and NCED1 were 0.998***, 0.978***, 0.982***, 0.956***, 0.986*** and 0.953***, respectively, whereas those of ABA-hy3, LEA D-34, GA2-ox8 and LeMADS ranged from 0.64 to 0.83 and were not significant (see Additional file 10)

To validate the results from the transcriptome analysis, DEGs related to the effect of rinsing with water in the germination were determined by qRT-PCR. We selected ten DEGs with putative functions related to ABA biosynthesis, catabolism and signaling; GA biosynthesis and catabolism; and stress response genes (see Additional file 8). These genes were highly significantly upregulated genes in KEGG and DEGs analysis. Specific primers were designed and optimized using PCR for the selected DEGs and for ubiquitin as the endogenous control. We amplified cDNA from five samples: BR, after rinsing 2 days (2D), after rinsing 7 days (7D), 2D4W and 7D4W (Fig. 3). The transcripts of stress response genes, such as *EID1*, *DREB2C* and *LEA D-34*, accumulated during germination and the expressions of most genes were upregulated in 2D and 7D, but downregulated in 7D4W (Fig. 3a, b and c). In particular, *LEA D-34* showed high expression in BR and followed by a dramatic declined. Most ABA biosynthetic genes were downregulated slightly in 7D4W (Fig. 3d, e and f), which was consistent with ABA contents in the both embryonic axes (see Additional file 9). The expressions of *ABA-hy3* and *PP2CA* in the embryonic axis of peach seeds were upregulated in 2D and 2D4W, and downregulated in 7D4W (Fig. 3g and h). Similarly, *GA2-ox8* produces inactive GA in GA biosynthesis [36] that was downregulated in 7D4W (Fig. 3i). *LeMADS* was upregulated in 2D and 7D, and significantly downregulated after chilling for 4 weeks (Fig. 3j). Analysis of transcript levels by qRT-PCR showed that the expressions of the ten selected genes corresponded with the differential expression patterns determined from the transcriptome analysis in 2D4W vs. 7D4W treatment (Fig. 4). There were significant relationships between the log (relative genes expression) and log (RPKM) for *EID1*, *DREB2C*, *AFP2*, *AFP3*, *PP2CA* and *NCED1* (see Additional file 10). The highly significant correlation of linear regression analysis, which indicated good reproducibility between transcript abundance assayed by RNA-seq and the expression profile revealed by qRT-PCR data. Although the data other four genes did not show a clear correlation between RNA-seq and qRT-PCR, the trends by the treatments were consistent for the ten genes.

## Discussion

### Genes associated with phytohormones

Phytohormones, such as ABA and GA, play an important role in controlling seed dormancy and germination [35, 37, 38], and mediate responses to both biotic and abiotic stresses [39]. ABA is a positive regulator of dormancy and a negative regulator of germination, whereas GA promotes seed germination [35]. In particular, ABA has been termed as ‘dorman’ or ‘dormancy inductor’ [40].

In this study, pathway analysis of KEGG analysis showed upregulation in 2D4W of the unigenes encoding proteins involved in ABA biosynthesis and catabolism, including *NCED1, ABA 8′-hydroxylase* in carotenoid biosynthesis and *PP2C* in plant hormone transduction pathways (see Additional file 6). While only *NCED1* and *PP2C* showed upreguration in BR. *NCED* is the key enzyme in ABA biosynthesis in higher plants [16], while ABA 8′-hydroxylase is the key enzyme in the oxidative catabolism of ABA and is expressed throughout fruit development [17]. The expression of *NCED1* is closely associated with the ABA level in imbibing dormant seeds of *Brachypodium distachyon* [41]. Our data suggested that these genes could be involved in modulation of embryo dormancy through ABA synthesis and catabolism.

ABA signaling is important for stress responses, seed development and dormancy [42, 43]. The activity of *PP2C* is controlled by ABA receptors, which upon binding to ABA form a complex with, and inactivate PP2C. Inactivation of PP2C leads to upregulation of *SnRK2*, which activates downstream transcription factors, including the ABA responsive element (ABRE) binding factor (ABF), *ABA insensitive5* (*ABI5*)*, ABI3* and *ABI4*, and thereby mediates seed responsiveness to ABA [44]. Twenty-one DEGs were annotated as probable *PP2C* and were upregulated in the endodormancy state in flower bud of Japanese pear [45]. In sorghum, the higher expression of an ABI5 homolog has been associated with higher levels of dormancy [46]. In this study, KEGG pathway analysis showed that transcripts of a gene encoding PP2C were significantly higher in BR and 2D4W. Similarly, the qRT-PCR results showed upregulation of *PP2C* in BR and 2D4W and dramatic downregulation in 7D4W (Fig. 3h). In addition, *ABI5-binding protein* genes were also significantly upregulated in 2D4W and significantly downregulated in 7D4W (Fig. 3e and f).

These data suggested that the ABA signaling pathway was still activate in dry seed and 2D4W; ie short period rinsing, and it might be significant for maintaining ABA sensitivity and endodormancy of the embryo. Transcripts of genes encoding GA2-oxidase (GA2-ox) 8, which inactivates GA_1_, were significantly higher in 2D4W. GA catabolic genes such as *GA2ox* are important to control GA levels [47–50]. These results suggested that the ABA signaling pathway and GA inactivating genes play an important role in dormancy and germination and were downregulated by the longer rinsed treatment in ‘Yaguchi’ peach seeds.

### Genes associated with stress response

Seed development and maturation are accompanied by increased desiccation tolerance [51]. Seeds activate a series of mechanisms to respond to many biotic and abiotic stresses during germination when placed in water or their external environment changes. The EID1-like protein 3 (EDL3) shows high similarity to EID1 (Empfindlicher im dunkelroten Licht 1), an F-box protein [52]. EID1 functions as a positive regulator in ABA-dependent signaling cascades that control seed germination, root growth, greening of etiolated seedlings and transition to flowering in *Arabidopsis thaliana* [52]. *EDL3* expression was induced under osmotic stress, high salinity, and ABA application [53]. Our results showed that rinsing for 7D was effective to decrease the expression of *EID1* gene both at 7D and 7D4W (Fig. 3a), indicating that longer rinsing decreased *EID1* expression.

Dehydration-responsive element-binding proteins (DREBs) are transcriptional regulators of the APETALA2/Ethylene Responsive element-binding Factor (AP2/ERF) family that control the expressions of abiotic stress-related genes. Under conditions of mild heat stress, transgenic seeds overexpressing *DREB2C* showed delayed germination and increased ABA content compared with the untransformed wild-type in *Arabidopsis* [54]. In this study, the DREB2CA loci were upregulated in BR, 2D and 2D4W (Fig. 3b), but downregulated in 7D4W, suggesting that 7D4W released seeds from endodormancy.

Furthermore, these results showed that transcripts of genes encoding the LEA D-34 protein or dehydrin (related to drought stress) there was higher expression of the genes encoding the LEA D-34 in BR and then downregulated after chilling (see Additional file 4 and Fig. 3c). LEA proteins are classified into at least five groups by the similarity in their amino acid sequences [55, 56] and are associated with dormancy transition [57]. In the Norway spruce (*Betula pubescens Ehrh*.), the expression of certain dehydrin genes gradually decreased when approaching bud burst [58]. In dormant Japanese pears, *LEA* genes are significantly higher in the deepest endodormancy period [45]. Additionally, dehydrins are synthesized by cells in response to ABA [59] and most *PmLEA* genes are upregulated by ABA treatment [60]. These results indicated that the *LEA* gene was induced to develop tolerance against drought in dry seeds without rinsing.

### Genes associated with transcription factors (TFs)

Transcription factors (TFs) play a crucial role in plant development and stress response [61]. One group of well-studied transcription factors involved in chilling responses is the AP2-EREBP family members, which have been subdivided into four major subfamilies: the DREB/CBF, AP2, ERF subfamilies [62]. Of these, the DREB/CBF subfamily has been reported to play a major role in the early stages of the chilling response [63], as evidenced by studies in numerous species such as Anthurium [64] and rice [65], as well as tea plant [66, 67]. In the GO molecular function and biological process categories, sequence-specific DNA binding TF activity and regulation of transcription were significantly enriched in 2D4W compared with 7D4W (Fig. 2). In other words, 7D4W, ie, a longer rinsing treatment, downregulated the expression of several TFs, such as heat stress TF, AP2-like ethylene-responsive TF, DREB2C, ethylene-responsive TF (ERF TF) and the MYB TF family (see Additional file 4). These results suggested that genes of sequence-specific DNA binding TF activity are associated with the effects of the longer rinsing combined with chilling on breaking dormancy in ‘Yaguchi’ seeds.

MADS-box TFs play fundamental roles in plant development, such as floral organ and meristems identity determination and transition from vegetative to reproductive growth regulation. MADS-box TFs are involved in bud dormancy regulation in peaches and other species [68–71]. *PpMADS13-1* and −*2*, isolated from the leaf bud of Japanese pear, appear to be upregulated towards endodormancy establishment and downregulated concomitant with endodormancy release [72, 73], similar to the peach *DAM5* and *DAM6* genes [74]. In this study, chilling treatment effectively reduced the expression of *LeMADS* (Fig. 3j). Similarly, the expressions of *DAM1* and *DAM6* were decreased significantly by chilling treatment in peach seeds [75]. These results indicated that MADS-box genes maintain seed dormancy and are repressed when seed dormancy is released by chilling.

## Conclusions

This study indicated that longer rinsing and chilling affects gene expression and germination rate, and investigated key seed dormancy-related genes using next-generation sequencing to profile the transcriptomes of dormant ‘Yaguchi’ peach seeds. We identified genes associated with the effects of the rinsing and chilling process, including genes associated with phytohormones, the stress response and TFs. The 7D4W treatment downregulated genes involved in ABA synthesis; *NCED1,* catabolism; *ABA 8′-hydroxylase*, ABA signaling pathway; *PP2C* and *ABI5* and GA inactivation; *GA2-ox8*, which eventually suppressed ABA activity and consequently promoting germination and seedling growth. Stress response genes (*EID1, DREB2CA* and *LEA D-34*) were also downregulated by 7D4W treatment, suggesting that it released seeds from endodormancy. TF genes such as *AP2-like ethylene-responsive, DREB2Cs, ERF* and *LeMADS* were upregulated in BR and 2D4W, suggesting these TFs play important roles in maintaining seed dormancy.

## Methods

### Plant materials

Fully ripened ‘Yaguchi’ peaches were collected from the peach flower garden of Koga city, Ibaraki prefecture, Japan, in middle October 2013 (36°10'N, 139°42'E). Voucher specimens were gathered from the leaf and seed specimens (Voucher specimen accession number: PPKYL0716-1; PPKYS0716-1) and identified by M. Yoshida and K. Yamane. After the skin and flesh were removed, endocarps were carefully cracked in a vice to remove the seeds. We hypothesized that longer rinsing combined with chilling of seeds can alter the genes expression in related to dormancy and then raise the germination rate in this peach. To prove this assumption, Seeds were rinsed for 2 or 7 days under running tap water and transferred into petri dishes containing two-layers of filter paper moistened with sterile water. The plastic petri dishes were sealed by parafilm and kept in the refrigerator at 5 °C for 2, 4, 6, and 8 weeks. The germination and height of seedlings were noted after 35 days. Three biological replicates of 10 embryonic axes of dry seed before and after rinsing and chilling were immediately frozen with liquid nitrogen and stored at −80 °C. For RNA extraction and qRT-PCR, BR; 2D; 7D; 2D4W and 7D4W were used. For library preparation, the sample from only BR, 2D4W and 7D4W were constructed due to these treatments were significantly different of germination rate (Fig. 1). Therefore, we would like to know how to difference of the differentially expression genes among these treatments.

### RNA isolation

Samples of 10 mg of frozen plant tissue were ground in liquid nitrogen to a fine powder. Total RNA was extracted from these tissues using the method described by [76]. Ten embryonic axes were extracted with three replications. RNA integrity was evaluated with a 1.0 % agarose gel stained with ethidium bromide. Then, total RNA were quantified and examined for protein contamination (A_260_/A_280_) and reagent contamination (A_260_/A_230_) by using a NanoDrop ND-1000 spectrophotometer. The RNA integrity number determined by the Agilent Technologies 2100 Bioanalyzer (Agilent Technologies) together with high sensitivity DNA Lab chip kit [77]. High quality total RNA samples give two distinct peaks and yield an RNA Integrity Number (RIN) value greater than 8. Total RNA (200 ng) from each biological triplicate was used for library preparation and sequenced.

### Library preparation and Illumina sequencing

The libraries for RNA-Seq were constructed using a SureSelect Strand-Specific RNA Library prep kit (Agilent Technologies), according to manufacturer's instructions. Oligo-(dT) magnetic beads were used to isolate poly-(A) mRNA from total RNA, and mRNA was fragmented in fragmentation buffer. Using these short fragments as templates, random hexamer primers were used to synthesize first-strand cDNA. Second-strand cDNA was synthesized using buffer, dNTPs, RNaseH, and DNA polymerase I. The product will be double-standed cDNA (ds cDNA). ds cDNA fragments were purified from free nucleotides, enzymes, buffers, and RNA with a AMPure XP beads (Beckman Coulter Genomics). Perform end-repair on purified eluted ds cDNA and adding poly (A), then ligated to sequencing adapters. Adaptors were ligating to both ends of the ds cDNA. After purification via AMPure XP beads again, suitable fragments were enriched by polymerase chain reaction (PCR) amplification and index the adaptor-ligated cDNA library. Each adaptor had eight-nucleotide difference in adaptor sequenced. A different index for each library reaction was use allowed for pooling libraries later for sequenced. Finally, sent normalized and pooled libraries to sequencing facility for cluster generation on a MiSeq Sequencer (Illumina, USA), the paired-end library was prepared following the protocol of the Illumina MiSeq Reagent Kits v3 (150 cycles, paired-end). All three replicates of the BR, 2D4W and 7D4W samples were sequenced.

### Bioinformatics analysis

#### Pre-processing of raw short read sequences and de novo assembly

Quality of sequence reads was checked with FastQC v0.11.5. Raw reads were first processed using cutadapt version 1.8.1 [78] to remove adapter sequences, low quality ends (quality scores < 30) and the last 76^th^ base, while reads shorter than 50-bp were discarded. The high-quality short reads were assembled into unigenes using the Trinity program version: trinityrnaseq-2.0.6 [79]. rRNA sequences were excluded from the unigenes by removing sequences matched to the SILVA rRNA database by the megablast program [80]. The high-quality short reads were mapped to the rRNA-removed unigenes using Bowtie [81]. Then, to estimate the expression levels of the transcripts, the number of uniquely mapped reads for each unigene was competed and normalized to RPKM values (reads per kilobase per million mapped reads) using RSEM method [82]. The RPKM values were then compared pairwise as: BR/2D4W, BR/7D4W, and 2D4W/7D4W. To identify DEGs, the edgeR package [83] was used to compute the *p*-value and fold change. The *p*-value was used to identify genes expressed differentially between the paired treatments. Significantly DGEs were identified using a FDR (false discovery rate) threshold of ≤ 0.05 and a minimum two-fold change.

### Validation of *de novo* assembly

To verify the accuracy of *de novo* assembly and annotation, we mapped reads on our assemblies and the *Prunus_persica*_v2.0.a1.all transcripts [29]. Mapping statistics were obtained using Bowtie2 (V.2.2.5) package.

### Functional annotation

To predict the biological functions of unigenes, sequences were used for BLAST searches and annotation against an NCBI nr protein database (NCBI non-redundant sequence database). Consensus sequences were further aligned by BLASTX to protein databases such as Swiss-Prot (*E*-value cut-off was set at 1e-5) [32], KEGG and GO. KEGG pathway mapping for unigenes were done using the KEGG automatic annotation server (KAAS) [33]. Putative coding regions were extracted from Trinity transcripts using TransDecoder [84]. GO annotation of the transcriptome was performed using InterproScan [85].

### Quantitative gene expression analysis

To validate the mRNA abundance of 10 genes that were significantly regulated by rinsing and chilling during the RNA-seq analysis, qRT-PCR was performed. Total RNA was isolated from embryonic axes and cDNAs were generated from the RNA samples as those used for the RNA-seq experiment. For each sample, 1 μg of total RNA was used with the QuantiTect® SYBR® Green RT-PCR Kit, according to the manufacturer’s protocol (Agilent). The cDNA was stored at −80 °C until use in the qRT-PCR analysis. The qRT-PCR assay was performed with three technical replicates using the Fast SYBR Green Master Mix (Life Technologies, Roche) on a Lightcycler 96 Real Time PCR system in a total volume of 15 μL. The PCR cycle comprised one 600 s cycle at 95 °C, followed by 45 cycles at 94 °C for 15 s, 60 °C for 30 s and 72 °C for 30 s. All amplified products were subjected to melt curve analysis. A negative control without a cDNA template was run with all analyses to evaluate the overall specificity. The reference gene ubiquitin was used to normalize the total amount of cDNA in each reaction. This gene was stably expressed in peach [74]. Amplification efficiency and relative gene expression levels were calculated using the ΔΔ*C*_T_ and 2^-ΔΔ*C*^_T_ methods (*C*_T_; cycle threshold). The Δ*C*_T_ value of each gene was calculated by subtracting the *C*_T_ value of the endogenous control from the *C*_T_ value of the target gene. Gene-specific primers were designed using primer-BLAST [86] (see Additional file 11).

## Abbreviations

ABA, Abscisic acid; DEGs, Differentially expressed genes; FC, Fold Change; FDR, False Discovery Rate; GA, Gibberelin; GO, Gene ontology; KEGG, Kyoto Encyclopedia of Genes and Genomes; PE, Paired-end; qRT-PCR, quantitative real-time reverse transcription PCR; RNA-seq, RNA-sequencing; TF, Transcription Factor

## Additional files

Additional file 1: Effect of rinsing and chilling on height of seedling. The horizontal axis is peach seeds after distinct periods of chilling after rinsing for 2 days and 7 days (2D and 7D). Non-overlapping letters (a–b) indicate significant difference between treatments, based on ANOVA analysis and Multiple Range Tests procedure with a confidence level of 95 %. (PDF 80 kb) Additional file 2: Table S1. Mapping statistics of reads to the *de novo* transcriptome assemblies and the *Prunus_persica*_v2.0.a1.all transcripts obtained using Bowtie2 (V.2.2.5) package. (PDF 83 kb) Additional file 3: List of all set of the differentially expressed genes. Summary of all set data and annotated by Swiss-Prot between BR *vs* 2D4W, BR *vs* 7D4W and 2D4W vs. 7D4W. (XLSX 15168 kb) Additional file 4: List of subset of the differentially expressed genes. Summary of up and down-regulations of differentially expressed genes and annotated by Swiss-Prot and KEGG between BR *vs* 2D4W, BR *vs* 7D4W and 2D4W vs. 7D4W filtered based on *p*-value and an adjusted FDR of less than 0.05. (XLSX 2681 kb) Additional file 5: Figure of distribution of differentially expressed genes. The DEGs showed in red logFC > |1|, *p-value* of <0.05 and FDR ratio of < 0.05 for each gene in each pair-wise comparison of BR, 2D4W and 7D4W. The black dots indicate non-differentially expressed genes. (JPG 473 kb) Additional file 6: Figure of pathway annotation by KEGG. Pathway annotation of BR *vs* 2D4W, BR *vs* 7D4W and 2D4W vs. 7D4W based on KEGG. (PDF 850 kb) Additional file 7: GO enrichment analysis. Raw data of Enrichment of gene ontology terms in differentially expression sequences in *Prunus persica* (L.) Batsch. (XLSX 93 kb) Additional file 8: Candidate-gene annotation by Swiss-Prot database. Candidate-gene involved in biosynthesis and catabolism of ABA, GA and stress response genes. (PDF 95 kb) Additional file 9: Effect of rinsing and chilling on ABA content. Non-overlapping letters (a–b) indicate significant difference between treatments, based on ANOVA analysis and Multiple Range Tests procedure with a confidence level of 95 %. (JPG 164 kb) Additional file 10: Coefficient analysis. Comparison of a coefficient between gene expression ratios obtained from qRT-PCR and RNA-seq data. The qRT-PCR LN values (expression ratios; X-axis) were plotted against RPKM LN values (Y-axis). *, ** and *** indicates a significant difference at *p ≤ 0.05, ≤ 0.01* and *≤ 0.001* respectively. (PDF 232 kb) Additional file 11: Gene-specific primers for qRT-PCR. (PDF 182 kb)
