# Supplementary figures and images for: Transcriptome analysis of seed dormancy after rinsing and chilling in ornamental peaches (Prunus persica (L.) Batsch)
Source: BMC Genomics. 2016 Aug 8;17:575. doi: 10.1186/s12864-016-2973-y (PMC4977653; doi:10.1186/s12864-016-2973-y)

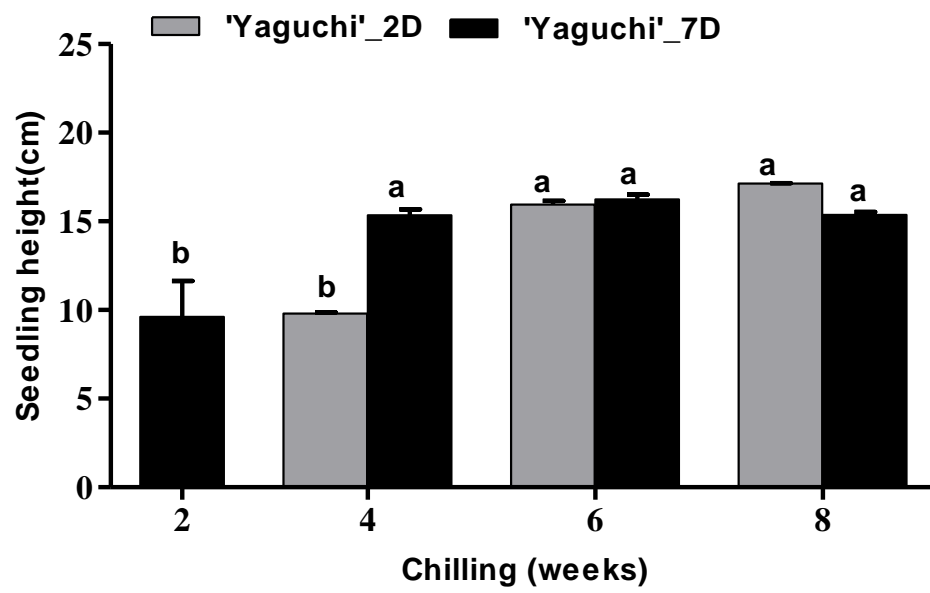

Supplement: Additional file 1: — Effect of rinsing and chilling on height of seedling. The horizontal axis is peach seeds after distinct periods of chilling after rinsing for 2 days and 7 days (2D and 7D). Non-overlapping letters (a–b) indicate significant difference between treatments, based on ANOVA analysis and Multiple Range Tests procedure with a confidence level of 95 %. (PDF 80 kb) [file 12864_2016_2973_MOESM1_ESM.pdf]

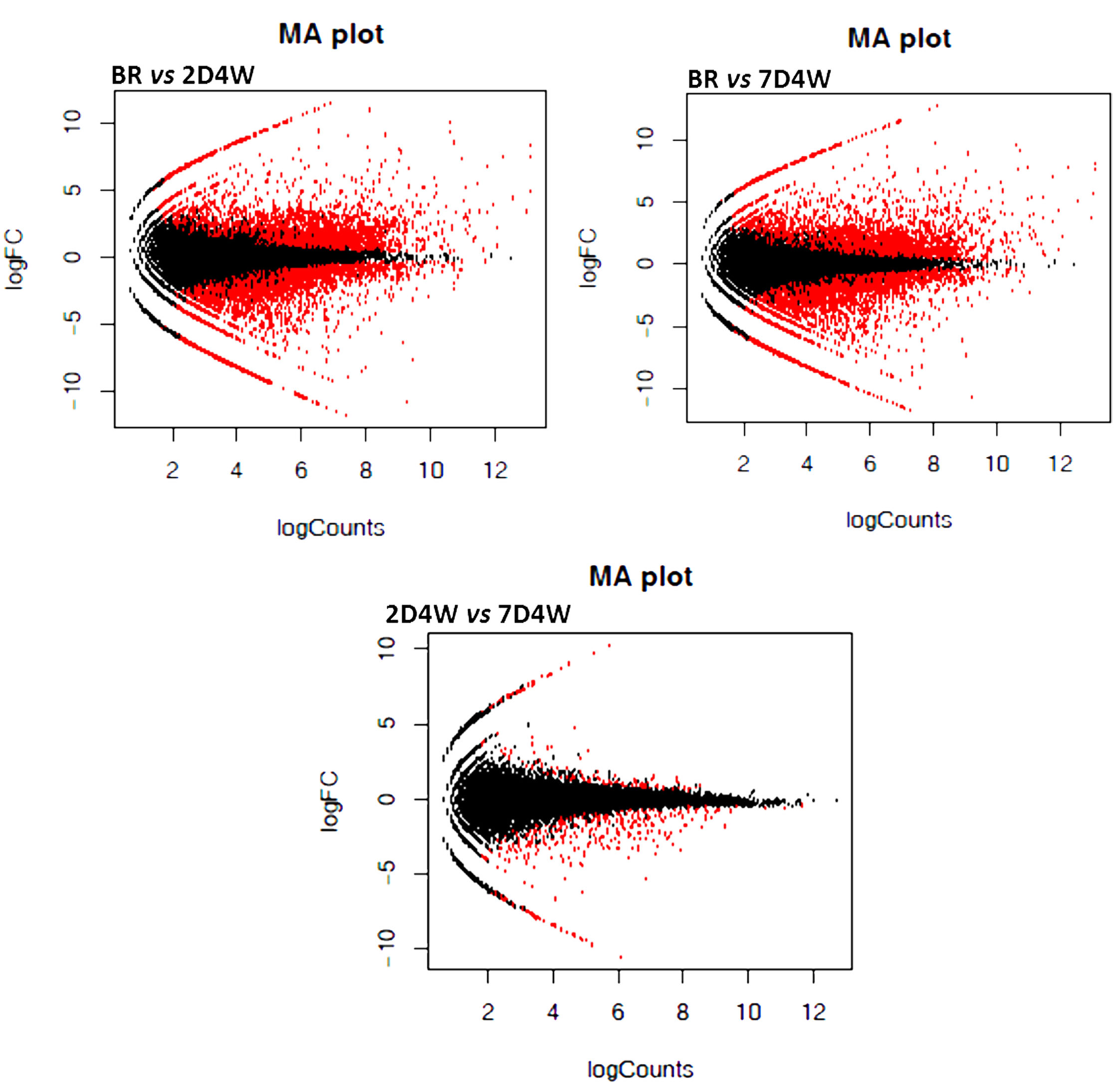

Supplement: Additional file 5: — Figure of distribution of differentially expressed genes. The DEGs showed in red logFC > |1|, p-value of <0.05 and FDR ratio of < 0.05 for each gene in each pair-wise comparison of BR, 2D4W and 7D4W. The black dots indicate non-differentially expressed genes. (JPG 473 kb) [file 12864_2016_2973_MOESM5_ESM.jpg]

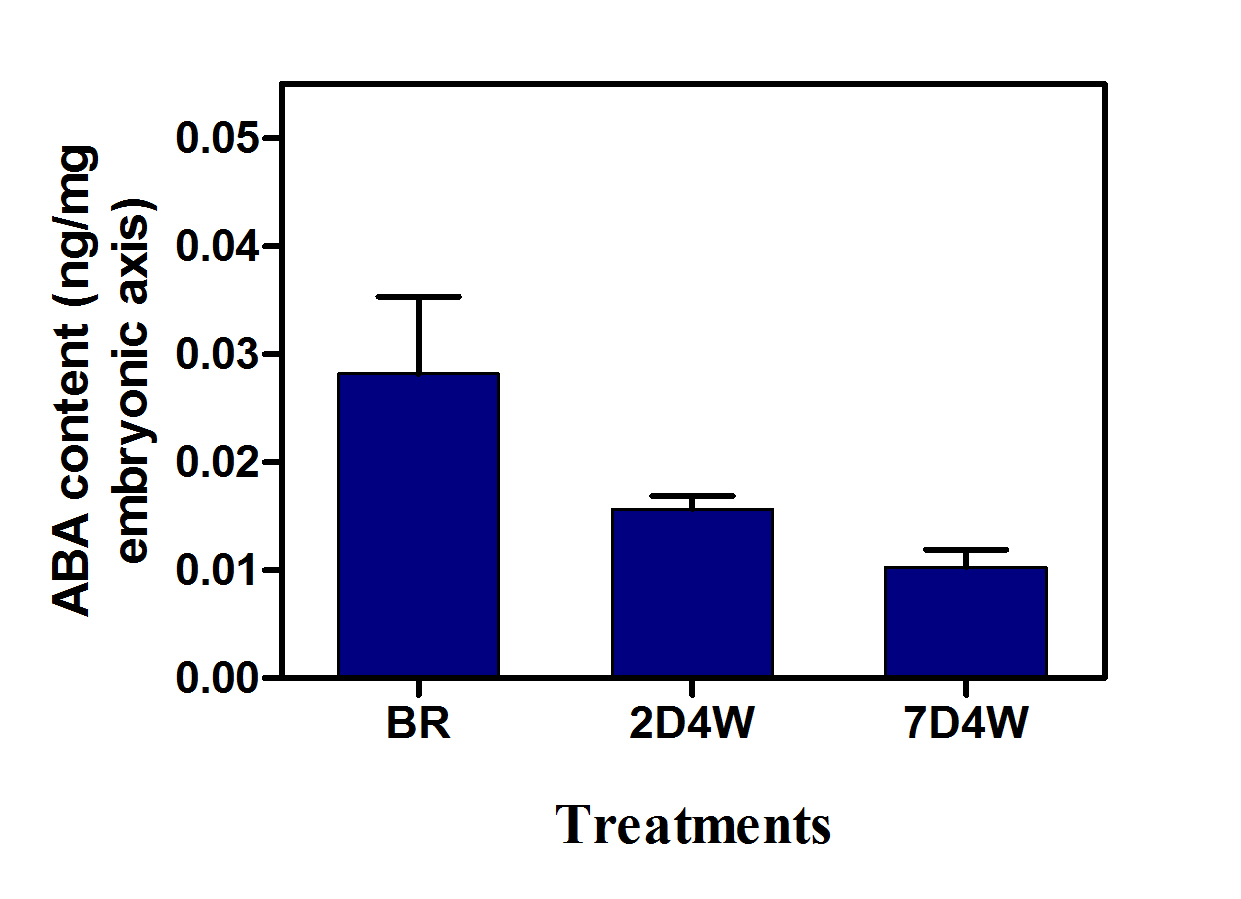

Supplement: Additional file 9: — Effect of rinsing and chilling on ABA content. Non-overlapping letters (a–b) indicate significant difference between treatments, based on ANOVA analysis and Multiple Range Tests procedure with a confidence level of 95 %. (JPG 164 kb) [file 12864_2016_2973_MOESM9_ESM.jpg]

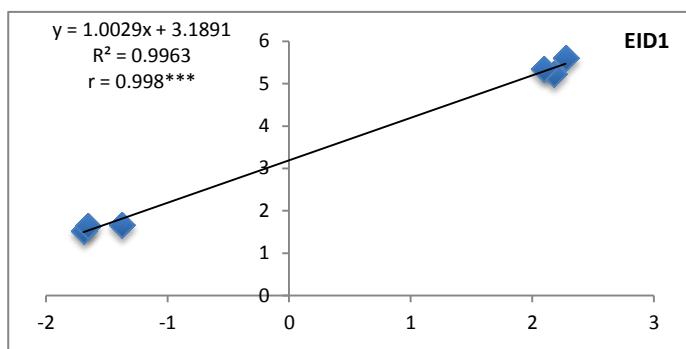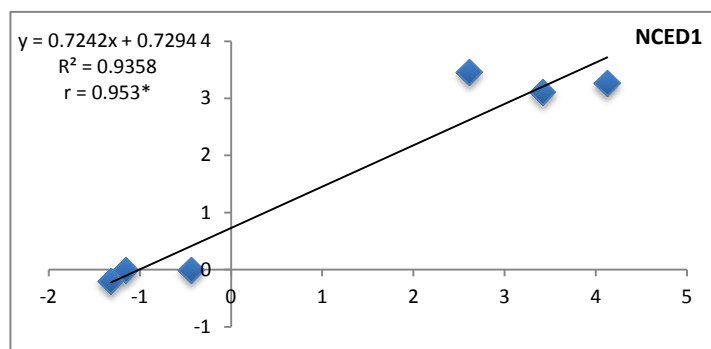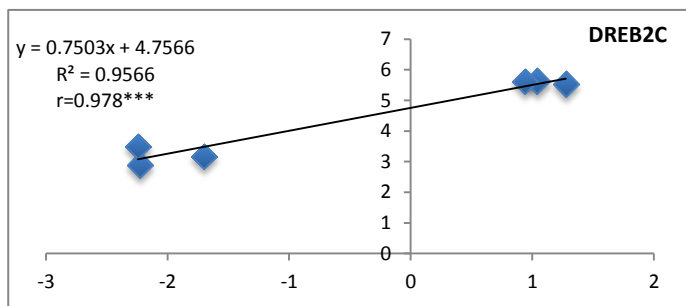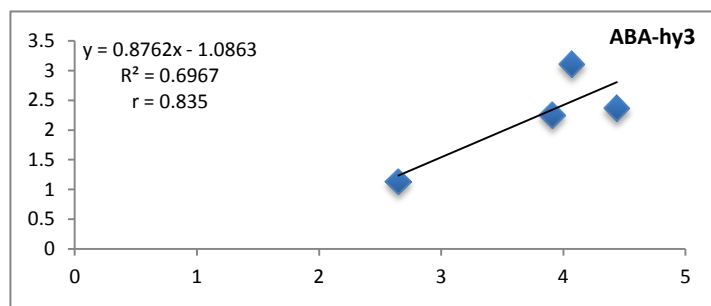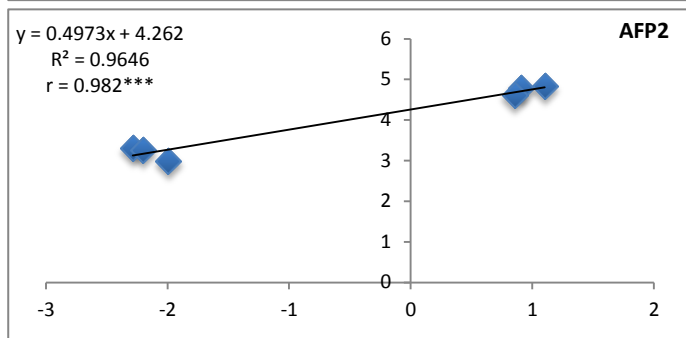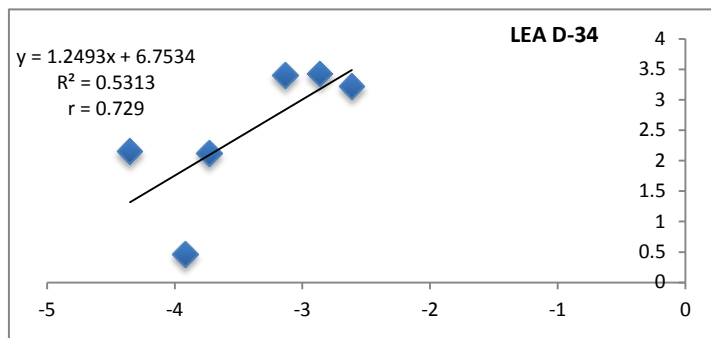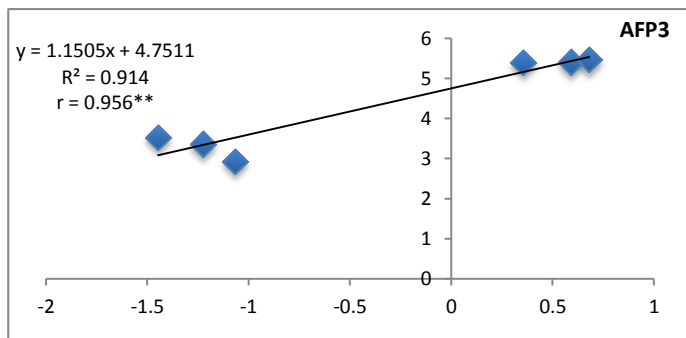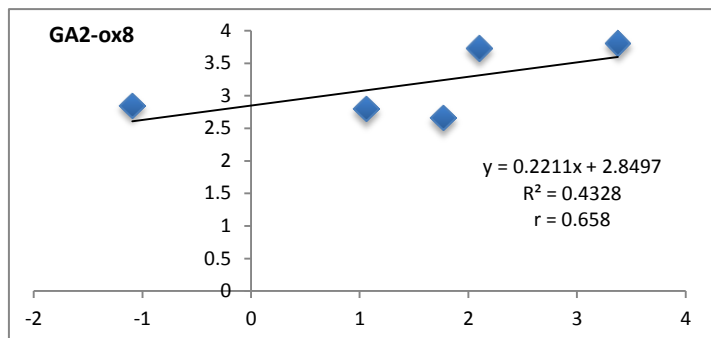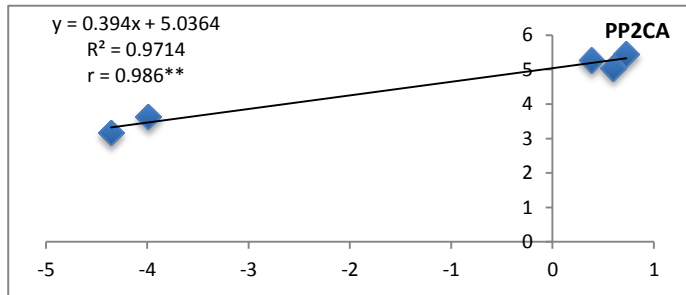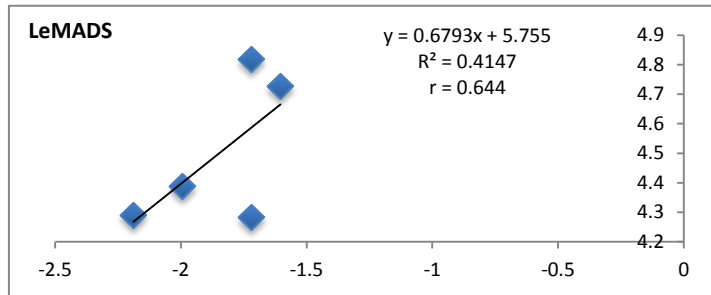

Supplement: Additional file 10: — Coefficient analysis. Comparison of a coefficient between gene expression ratios obtained from qRT-PCR and RNA-seq data. The qRT-PCR LN values (expression ratios; X-axis) were plotted against RPKM LN values (Y-axis). *, ** and *** indicates a significant difference at p ≤ 0.05, ≤ 0.01 and ≤ 0.001 respectively. (PDF 232 kb) [file 12864_2016_2973_MOESM10_ESM.pdf]
