# Supplementary material for: Transcriptome analysis of seed dormancy after rinsing and chilling in ornamental peaches (Prunus persica (L.) Batsch)
Source: BMC Genomics. 2016 Aug 8;17:575. doi: 10.1186/s12864-016-2973-y (PMC4977653; doi:10.1186/s12864-016-2973-y)
Supplement: Additional file 2: Table S1. — Mapping statistics of reads to the de novo transcriptome assemblies and the Prunus_persica_v2.0.a1.all transcripts obtained using Bowtie2 (V.2.2.5) package. (PDF 83 kb) [file 12864_2016_2973_MOESM2_ESM.pdf]

Table S1 Mapping statistics of reads to the *de novo* transcriptome assemblies and peach total transcript obtained using Bowtie2 (V.2.2.5) package.

Sample	Reads mapped to peach total transcript of *Prunus persica* annotation v2.1 (%)	Reads mapped to *de novo* assembly (%)
BR	75.85%	95.38%
2D4W	74.41%	95.42%
7D4W	75.17%	95.57%

Abbreviations: BR, dry seed before rinsing and chilling; 2D4W, seed rinsed 2 days and chilling 4 weeks; 7D4W, seed rinsed 7 days and chilling 4 weeks.
