# Supplementary material for: Transcriptome analysis of seed dormancy after rinsing and chilling in ornamental peaches (Prunus persica (L.) Batsch)
Source: BMC Genomics. 2016 Aug 8;17:575. doi: 10.1186/s12864-016-2973-y (PMC4977653; doi:10.1186/s12864-016-2973-y)
Supplement: Additional file 6: — Figure of pathway annotation by KEGG. Pathway annotation of BR vs 2D4W, BR vs 7D4W and 2D4W vs. 7D4W based on KEGG. (PDF 850 kb) [file 12864_2016_2973_MOESM6_ESM.pdf]

Enzymes involved in the abscisic acid biosynthesis based on KEGG. The enzymes that were found in this study were marked by red boxes. Gray boxes indicate the enzymes that were not found.

BR *vs* 2D4W; BR_UP

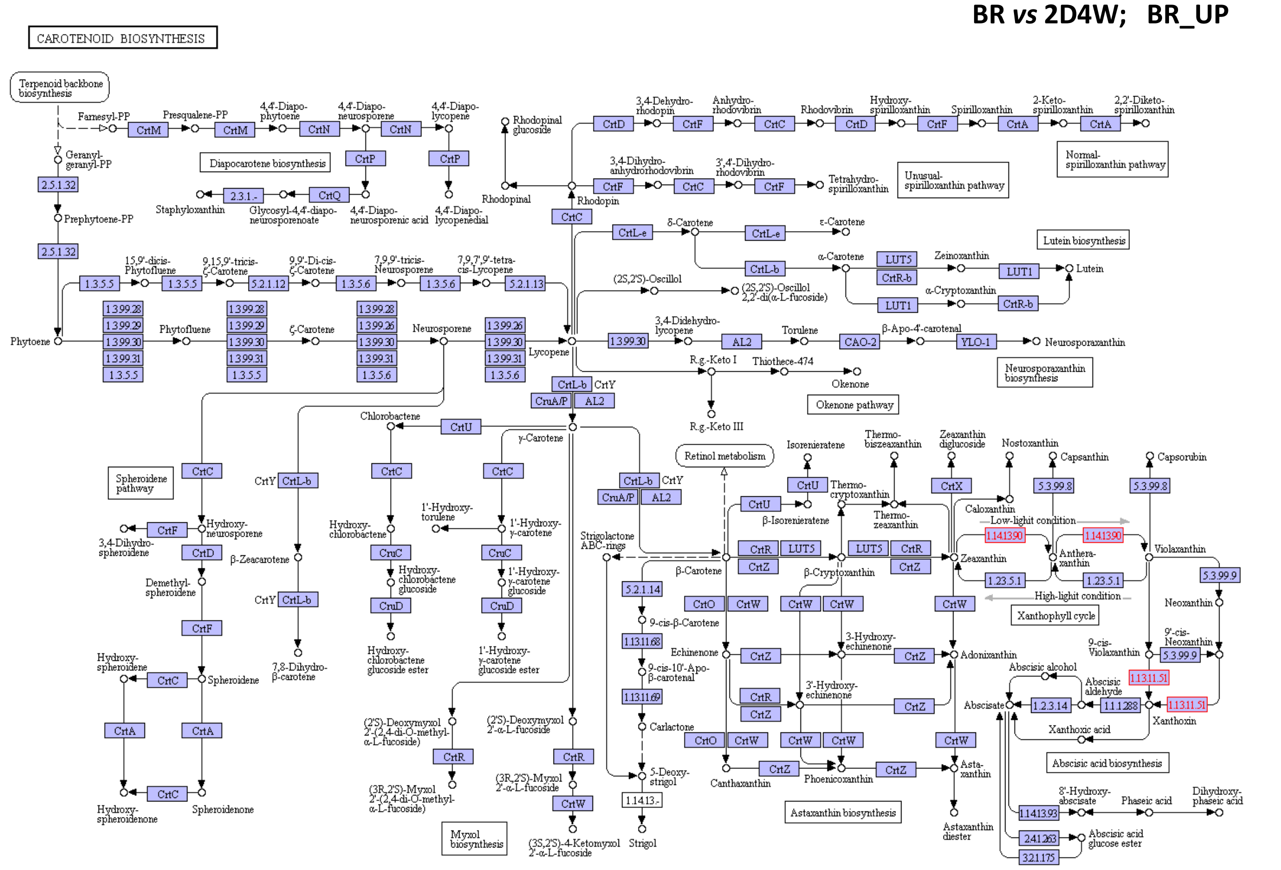

BR *vs* 2D4W; 2D4W_UP

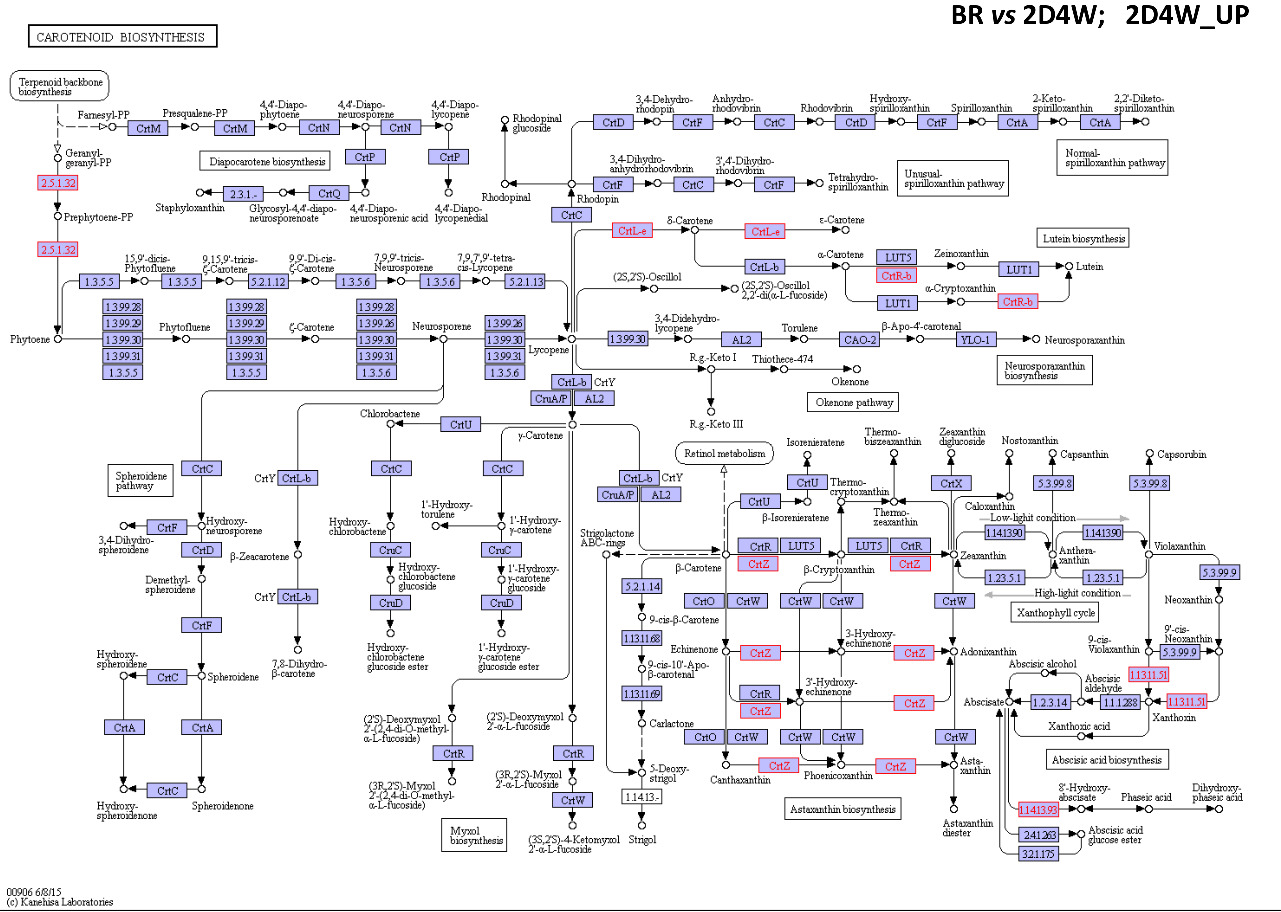

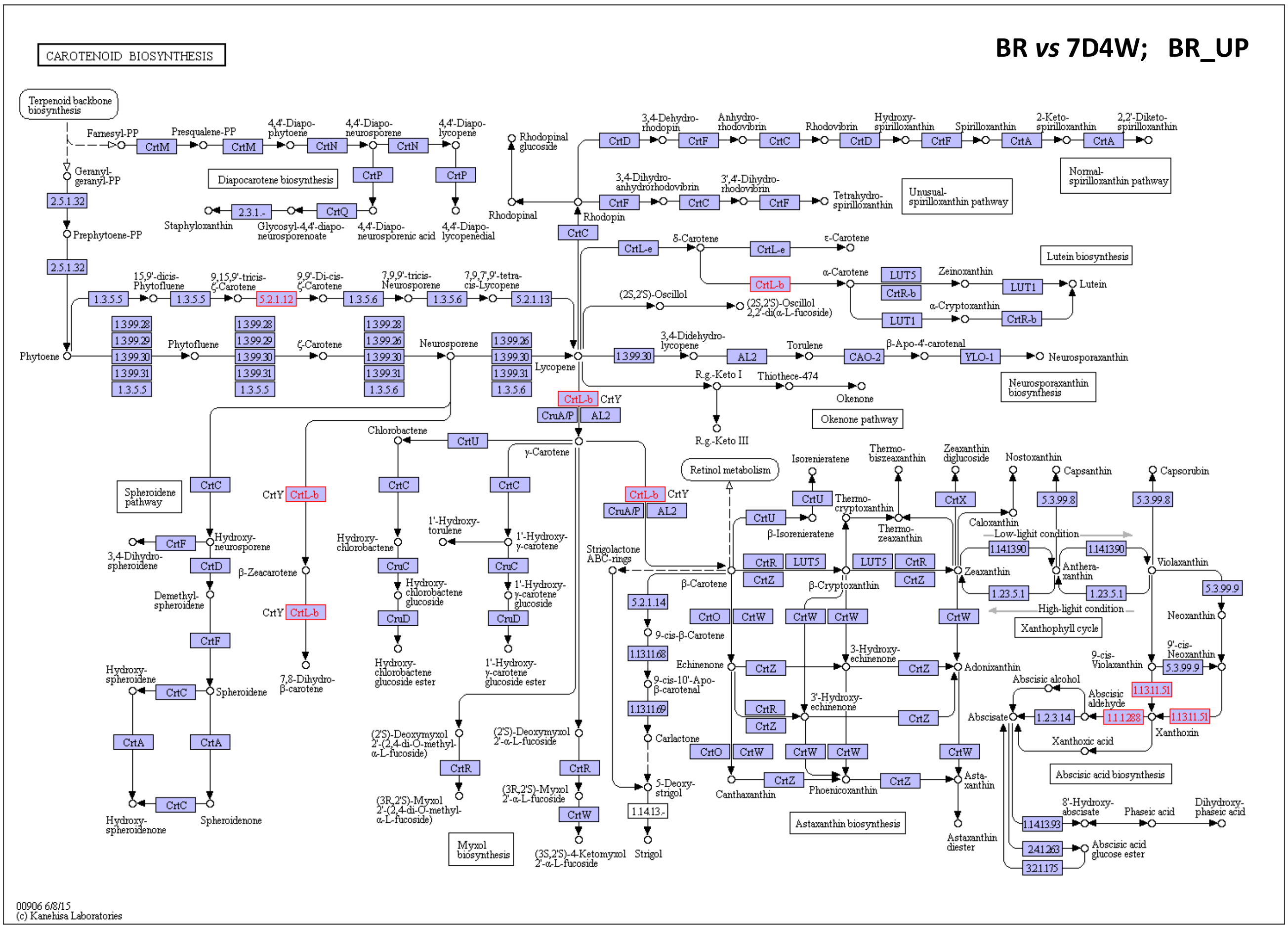
BR vs 7D4W; BR_UP
CAROTENOID BIOSYNTHESIS
Terpenoid backbone biosynthesis
Farnesyl-PP
Presqualene-PP
4,4'-Diapo-phytoene
4,4'-Diapo-neurosporene
4,4'-Diapo-lycopene
CrtM
CrtN
CrtP
CrtQ
Diapocarotene biosynthesis
Staphyloxanthin
Glycosyl-4,4'-diapo-neurosporenoate
4,4'-Diapo-neurosporenic acid
4,4'-Diapo-lycopenedial
Geranyl-geranyl-PP
2.5.1.32
Prephytoene-PP
15,9'-dicis-Phytofluene
9,15,9'-tricis-ζ-Carotene
9,9'-Di-cis-ζ-Carotene
7,9,9'-tricis-Neurosporene
7,9,7',9'-tetra-cis-Lycopene
1.3.5.5
5.2.1.12
1.3.5.6
5.2.1.13
1.3.99.28
1.3.99.29
1.3.99.30
1.3.99.31
1.3.99.26
Phytoene
Phytofluene
ζ-Carotene
Neurosporene
Lycopene
Rhodopinal glucoside
Rhodopinal
Rhodopin
3,4-Dehydro-rhodopin
Anhydro-rhodovibrin
Rhodovibrin
Hydroxy-spirilloxanthin
Spirilloxanthin
2-Keto-spirilloxanthin
2,2'-Diketo-spirilloxanthin
3,4-Dihydro-anhydrorhodovibrin
3',4'-Dihydro-rhodovibrin
Tetrahydro-spirilloxanthin
Normal-spirilloxanthin pathway
Unusual-spirilloxanthin pathway
CrtD
CrtF
CrtC
CrtA
CrtL-e
CrtL-b
δ-Carotene
ε-Carotene
α-Carotene
LUT5
CrtR-b
LUT1
Zeinoxanthin
α-Cryptoxanthin
Lutein
Lutein biosynthesis
(2S,2'S)-Oscillol
(2S,2'S)-Oscillol 2,2'-di(α-L-fucoside)
3,4-Didehydro-lycopene
AL2
Torulene
CAO-2
β-Apo-4'-carotenal
YLO-1
Neurosporaxanthin
Neurosporaxanthin biosynthesis
R.g.-Keto I
Thiothece-474
Okenone
Okenone pathway
R.g.-Keto III
CrtY
CruA/P
Chlorobactene
CrtU
γ-Carotene
Spheroidene pathway
Hydroxy-neurosporene
3,4-Dihydro-spheroidene
Demethyl-spheroidene
β-Zeacarotene
7,8-Dihydro-β-carotene
Hydroxy-spheroidene
Spheroidene
Hydroxy-spheroidenone
Spheroidenone
1'-Hydroxy-torulene
1'-Hydroxy-γ-carotene
Hydroxy-chlorobactene
Hydroxy-chlorobactene glucoside
1'-Hydroxy-γ-carotene glucoside
Hydroxy-chlorobactene glucoside ester
1'-Hydroxy-γ-carotene glucoside ester
CruC
CruD
(2'S)-Deoxymyxol 2'-(2,4-di-O-methyl-α-L-fucoside)
(2'S)-Deoxymyxol 2'-α-L-fucoside
(3R,2'S)-Myxol 2'-(2,4-di-O-methyl-α-L-fucoside)
(3R,2'S)-Myxol 2'-α-L-fucoside
(3S,2'S)-4-Ketomyxol 2'-α-L-fucoside
Myxol biosynthesis
CrtR
CrtW
Retinol metabolism
Strigolactone ABC-rings
β-Carotene
5.2.1.14
9-cis-β-Carotene
1.13.11.68
9-cis-10'-Apo-β-carotenal
1.13.11.69
Carlactone
5-Deoxy-strigol
1.14.13.-
Strigol
Isorenieratene
β-Isorenieratene
CrtO
CrtZ
β-Cryptoxanthin
Echinenone
3-Hydroxy-echinenone
3'-Hydroxy-echinenone
Canthaxanthin
Phoenicoxanthin
Astaxanthin
Astaxanthin diester
Astaxanthin biosynthesis
Adonixanthin
Thermo-biszeaxanthin
Thermo-cryptoxanthin
Thermo-zeaxanthin
Zeaxanthin diglucoside
Nostoxanthin
Caloxanthin
CrtX
Zeaxanthin
Antheraxanthin
Violaxanthin
1.14.15.21
1.23.5.1
Low-light condition
High-light condition
Xanthophyll cycle
Capsanthin
Capsorubin
5.3.99.8
5.3.99.9
Neoxanthin
9'-cis-Neoxanthin
9-cis-Violaxanthin
1.13.11.51
Xanthoxin
Abscisic aldehyde
1.1.1.288
Abscisic alcohol
Abscisate
1.2.3.14
Xanthoxic acid
Abscisic acid biosynthesis
8'-Hydroxy-abscisate
Phaseic acid
Dihydro-phaseic acid
1.14.13.93
2.4.1.263
3.2.1.175
Abscisic acid glucose ester
00906 6/8/15
(c) Kanehisa Laboratories

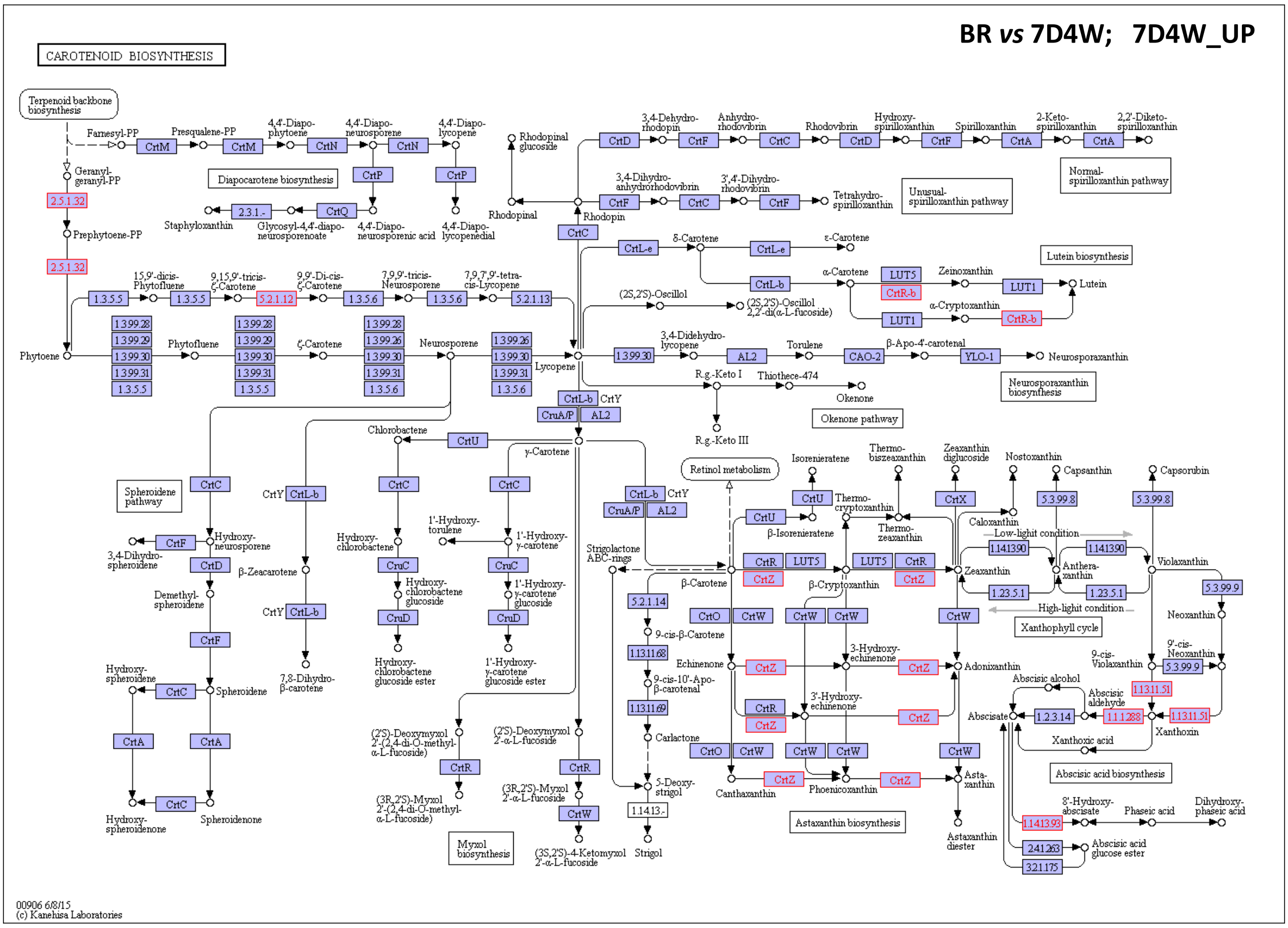
BR vs 7D4W; 7D4W_UP
CAROTENOID BIOSYNTHESIS
00906 6/8/15
(c) Kanehisa Laboratories

2D4W *vs* 7D4W; 2D4W_UP

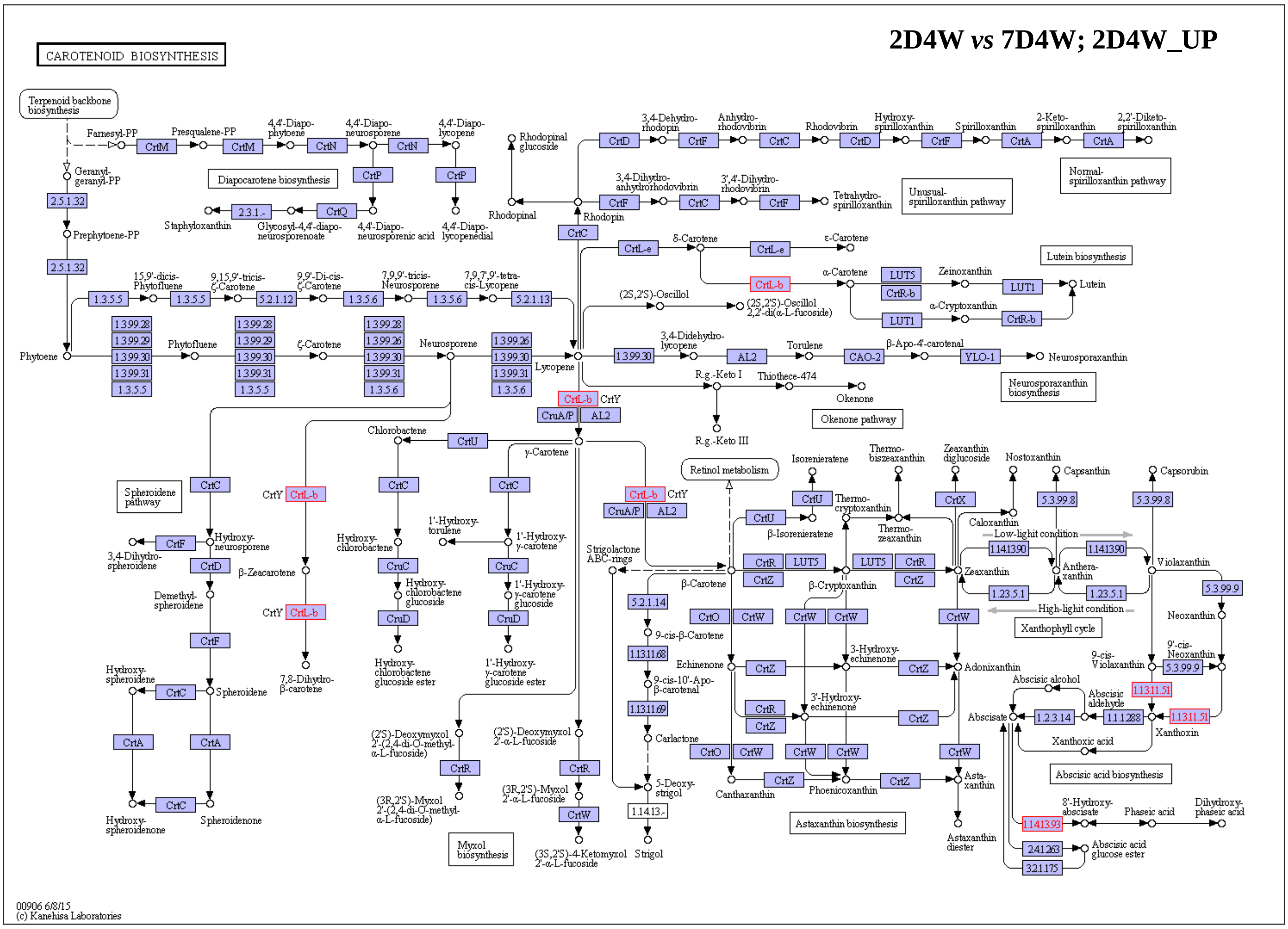

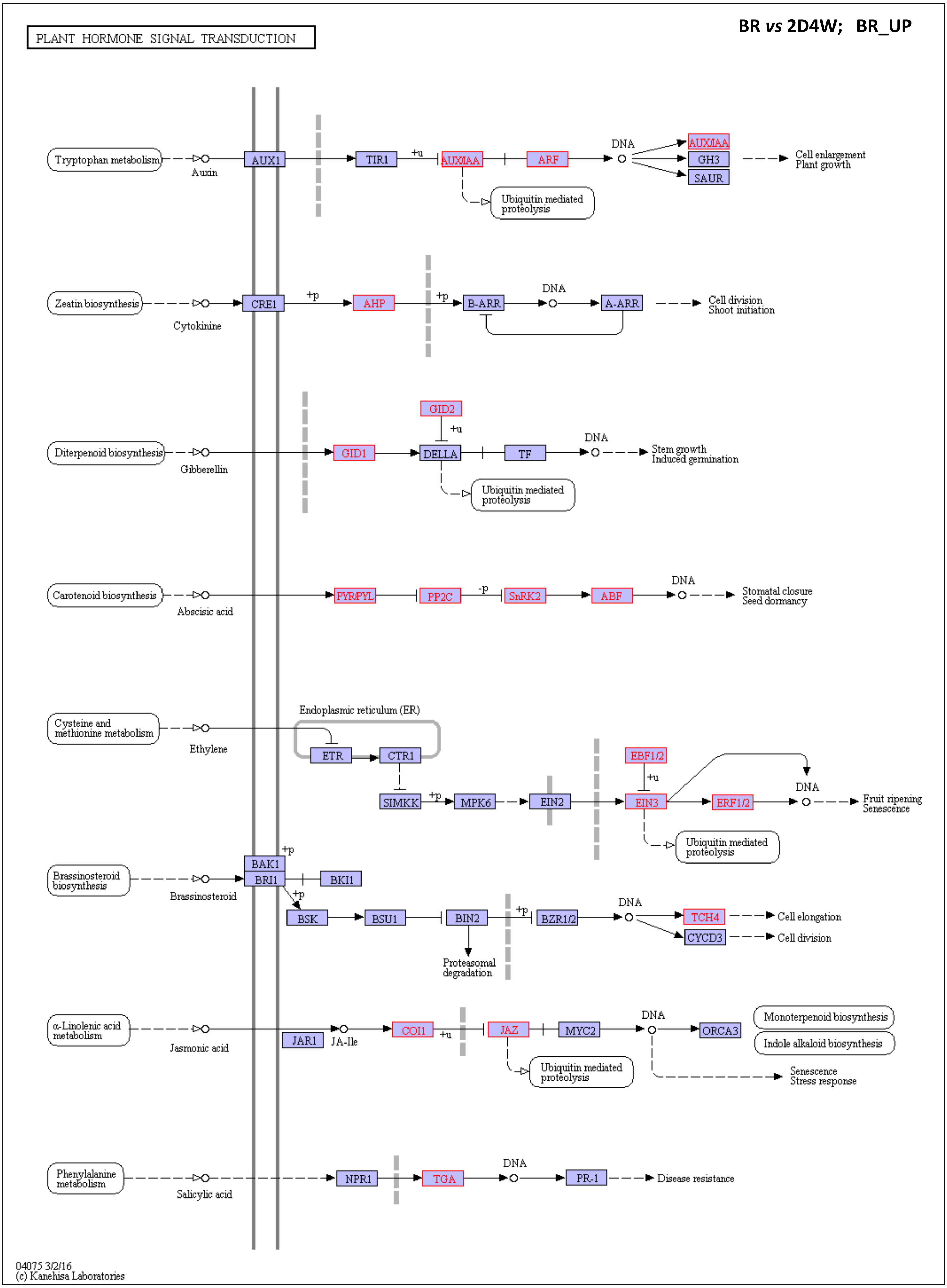

PLANT HORMONE SIGNAL TRANSDUCTION
BR *vs* 2D4W; BR_UP
Tryptophan metabolism
Auxin
AUX1
TIR1
+u
AUX/IAA
ARF
DNA
AUX/IAA
GH3
SAUR
Cell enlargement
Plant growth
Ubiquitin mediated proteolysis
Zeatin biosynthesis
Cytokinine
CRE1
+p
AHP
+p
B-ARR
DNA
A-ARR
Cell division
Shoot initiation
GID2
+u
Diterpenoid biosynthesis
Gibberellin
GID1
DELLA
TF
DNA
Stem growth
Induced germination
Ubiquitin mediated proteolysis
Carotenoid biosynthesis
Abscisic acid
PYR/PYL
PP2C
-p
SnRK2
ABF
DNA
Stomatal closure
Seed dormancy
Endoplasmic reticulum (ER)
Cysteine and methionine metabolism
Ethylene
ETR
CTR1
SIMKK
+p
MPK6
EIN2
EBF1/2
+u
EIN3
ERF1/2
DNA
Fruit ripening
Senescence
Ubiquitin mediated proteolysis
+p
BAK1
BRI1
BKI1
Brassinosteroid biosynthesis
Brassinosteroid
+p
BSK
BSU1
BIN2
+p
BZR1/2
DNA
TCH4
CYCD3
Cell elongation
Cell division
Proteasomal degradation
α-Linolenic acid metabolism
Jasmonic acid
JAR1
JA-Ile
COI1
+u
JAZ
MYC2
DNA
ORCA3
Monoterpenoid biosynthesis
Indole alkaloid biosynthesis
Ubiquitin mediated proteolysis
Senescence
Stress response
Phenylalanine metabolism
Salicylic acid
NPR1
TGA
DNA
PR-1
Disease resistance
04075 3/2/16
(c) Kanehisa Laboratories

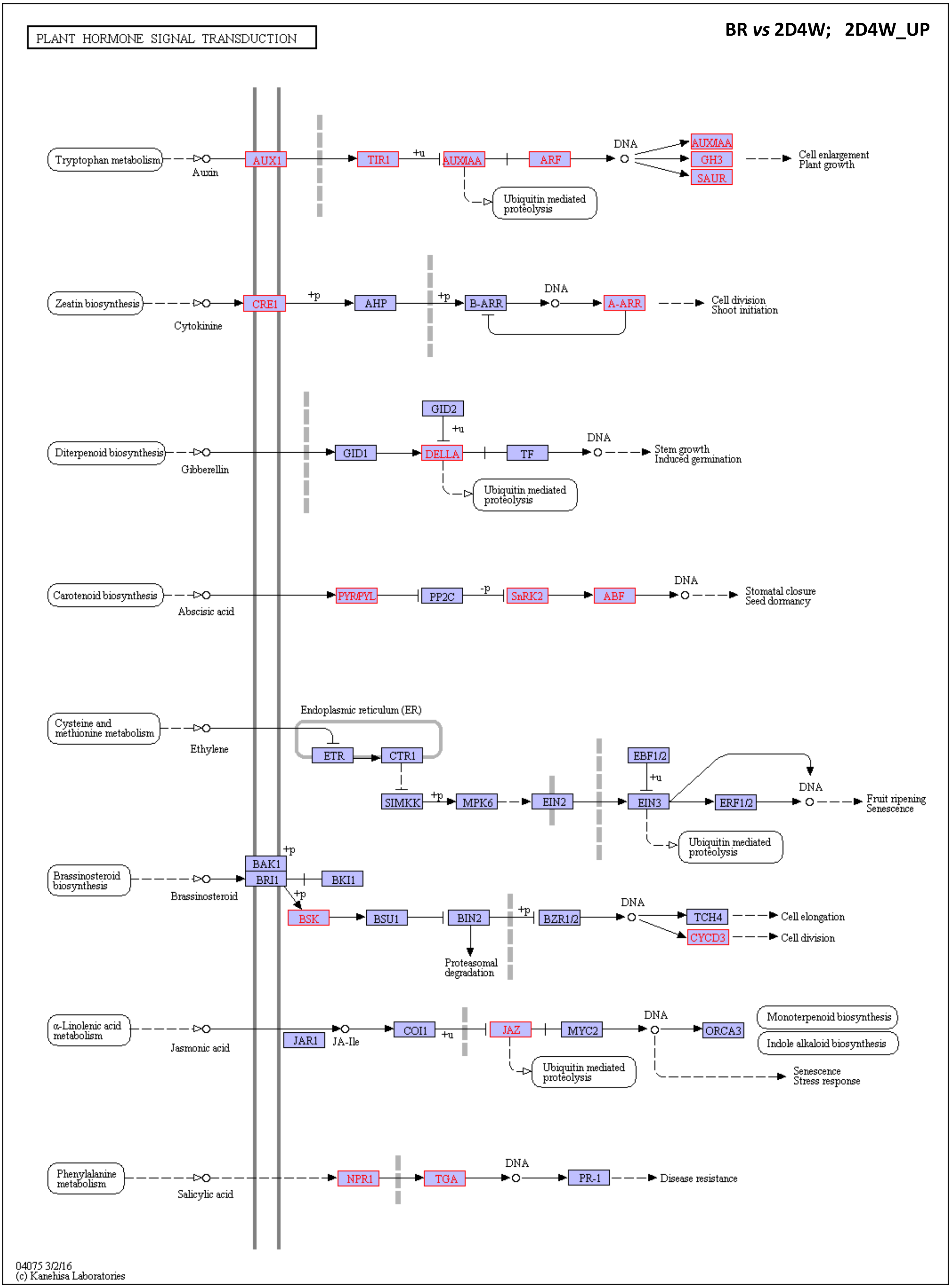
BR vs 2D4W; 2D4W_UP
PLANT HORMONE SIGNAL TRANSDUCTION
Tryptophan metabolism
Auxin
AUX1
TIR1
+u
AUX/IAA
ARF
DNA
AUX/IAA
GH3
SAUR
Cell enlargement
Plant growth
Ubiquitin mediated proteolysis
Zeatin biosynthesis
Cytokinine
CRE1
+p
AHP
+p
B-ARR
DNA
A-ARR
Cell division
Shoot initiation
Diterpenoid biosynthesis
Gibberellin
GID1
GID2
+u
DELLA
TF
DNA
Stem growth
Induced germination
Ubiquitin mediated proteolysis
Carotenoid biosynthesis
Abscisic acid
PYR/PYL
PP2C
-p
SnRK2
ABF
DNA
Stomatal closure
Seed dormancy
Cysteine and methionine metabolism
Ethylene
Endoplasmic reticulum (ER)
ETR
CTR1
SIMKK
+p
MPK6
EIN2
EBF1/2
+u
EIN3
ERF1/2
DNA
Fruit ripening
Senescence
Ubiquitin mediated proteolysis
Brassinosteroid biosynthesis
Brassinosteroid
BAK1
+p
BRI1
BKI1
+p
BSK
BSU1
BIN2
+p
BZR1/2
DNA
TCH4
CYCD3
Cell elongation
Cell division
Proteasomal degradation
α-Linolenic acid metabolism
Jasmonic acid
JAR1
JA-Ile
COI1
+u
JAZ
MYC2
DNA
ORCA3
Monoterpenoid biosynthesis
Indole alkaloid biosynthesis
Ubiquitin mediated proteolysis
Senescence
Stress response
Phenylalanine metabolism
Salicylic acid
NPR1
TGA
DNA
PR-1
Disease resistance
04075 3/2/16
(c) Kanehisa Laboratories

BR *vs* 7D4W; BR_UP

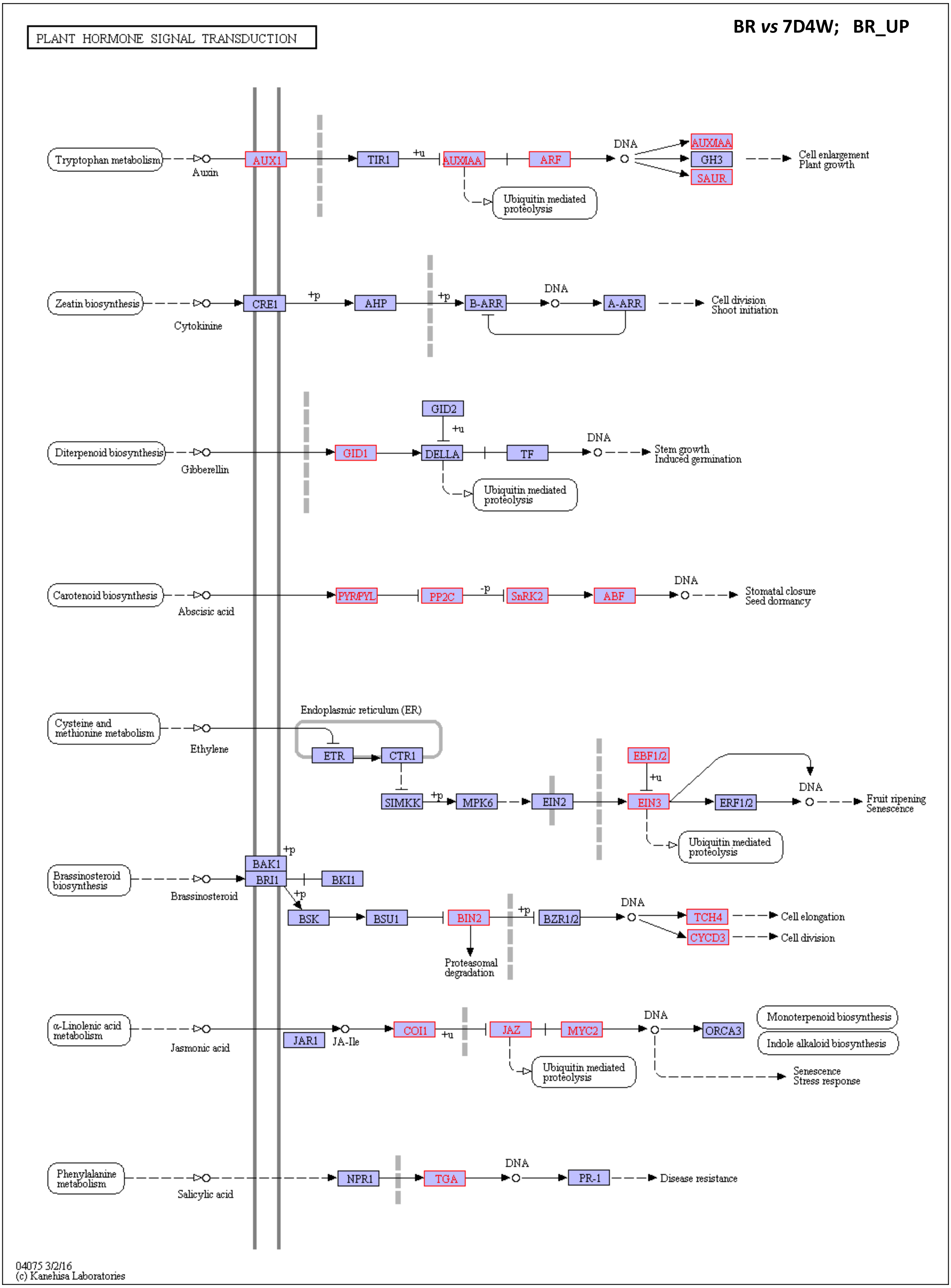

BR *vs* 7D4W; 7D4W_UP

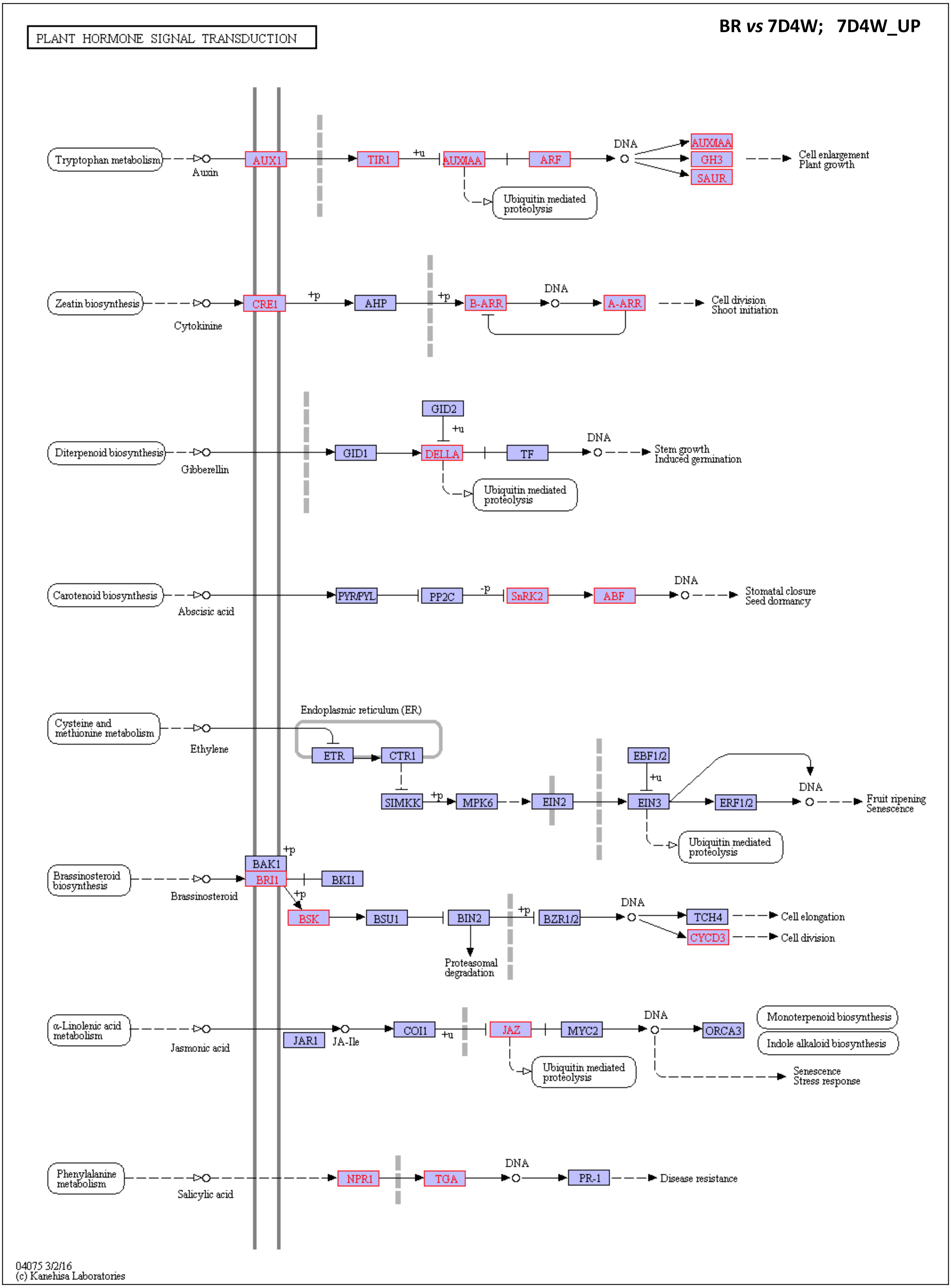

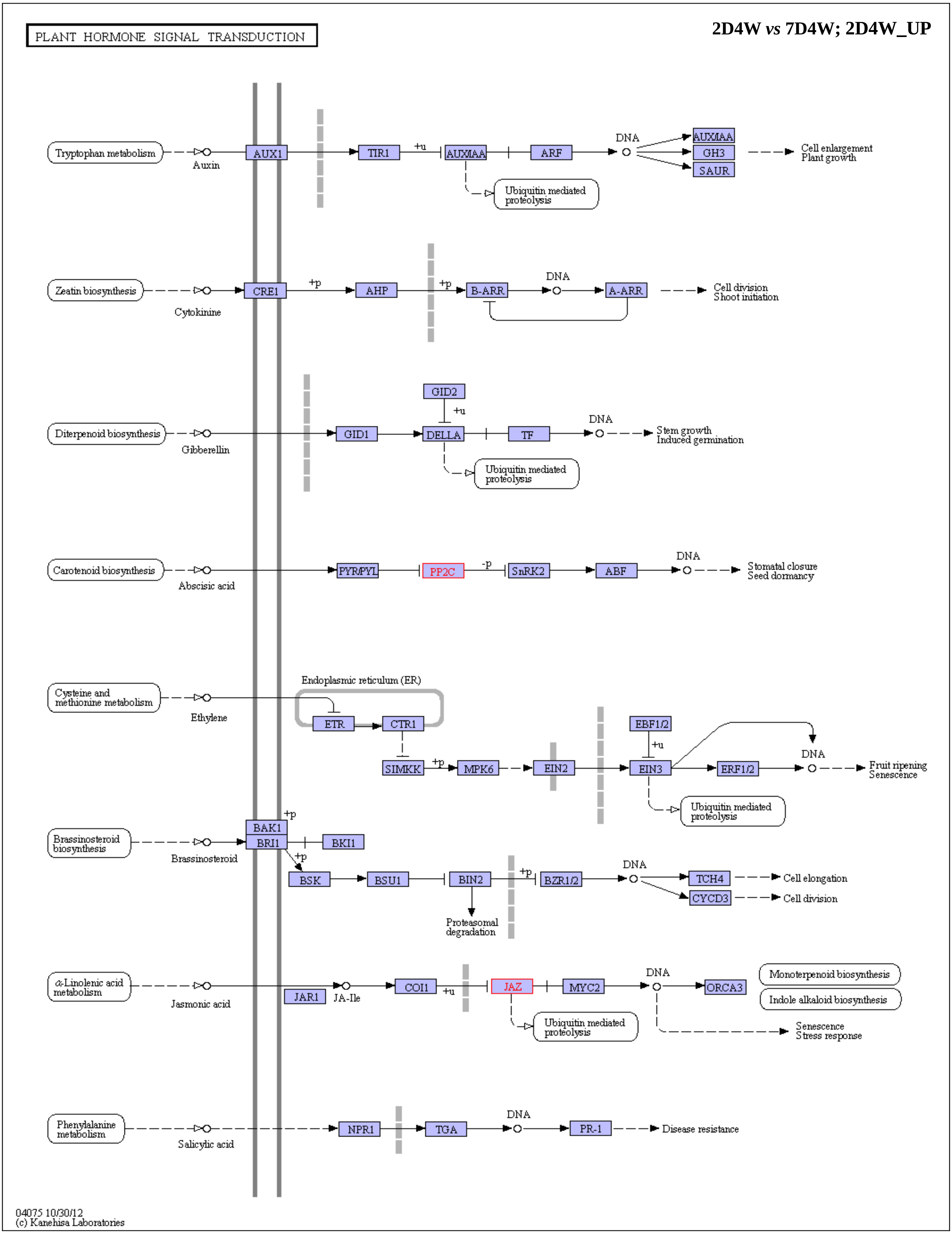

PLANT HORMONE SIGNAL TRANSDUCTION
2D4W vs 7D4W; 2D4W_UP
Tryptophan metabolism
Auxin
AUX1
TIR1
+u
AUX/IAA
ARF
DNA
AUX/IAA
GH3
SAUR
Cell enlargement
Plant growth
Ubiquitin mediated proteolysis
Zeatin biosynthesis
Cytokinine
CRE1
+p
AHP
+p
B-ARR
DNA
A-ARR
Cell division
Shoot initiation
GID2
+u
Diterpenoid biosynthesis
Gibberellin
GID1
DELLA
TF
DNA
Stem growth
Induced germination
Ubiquitin mediated proteolysis
Carotenoid biosynthesis
Abscisic acid
PYR/PYL
PP2C
-p
SnRK2
ABF
DNA
Stomatal closure
Seed dormancy
Endoplasmic reticulum (ER)
Cysteine and methionine metabolism
Ethylene
ETR
CTR1
EBF1/2
+u
SIMKK
+p
MPK6
EIN2
EIN3
ERF1/2
DNA
Fruit ripening
Senescence
Ubiquitin mediated proteolysis
BAK1
+p
Brassinosteroid biosynthesis
Brassinosteroid
BRI1
BKI1
+p
BSK
BSU1
BIN2
+p
BZR1/2
DNA
TCH4
CYCD3
Cell elongation
Cell division
Proteasomal degradation
α-Linolenic acid metabolism
Jasmonic acid
JAR1
JA-Ile
COI1
+u
JAZ
MYC2
DNA
ORCA3
Monoterpenoid biosynthesis
Indole alkaloid biosynthesis
Ubiquitin mediated proteolysis
Senescence
Stress response
Phenylalanine metabolism
Salicylic acid
NPR1
TGA
DNA
PR-1
Disease resistance
04075 10/30/12
(c) Kanehisa Laboratories

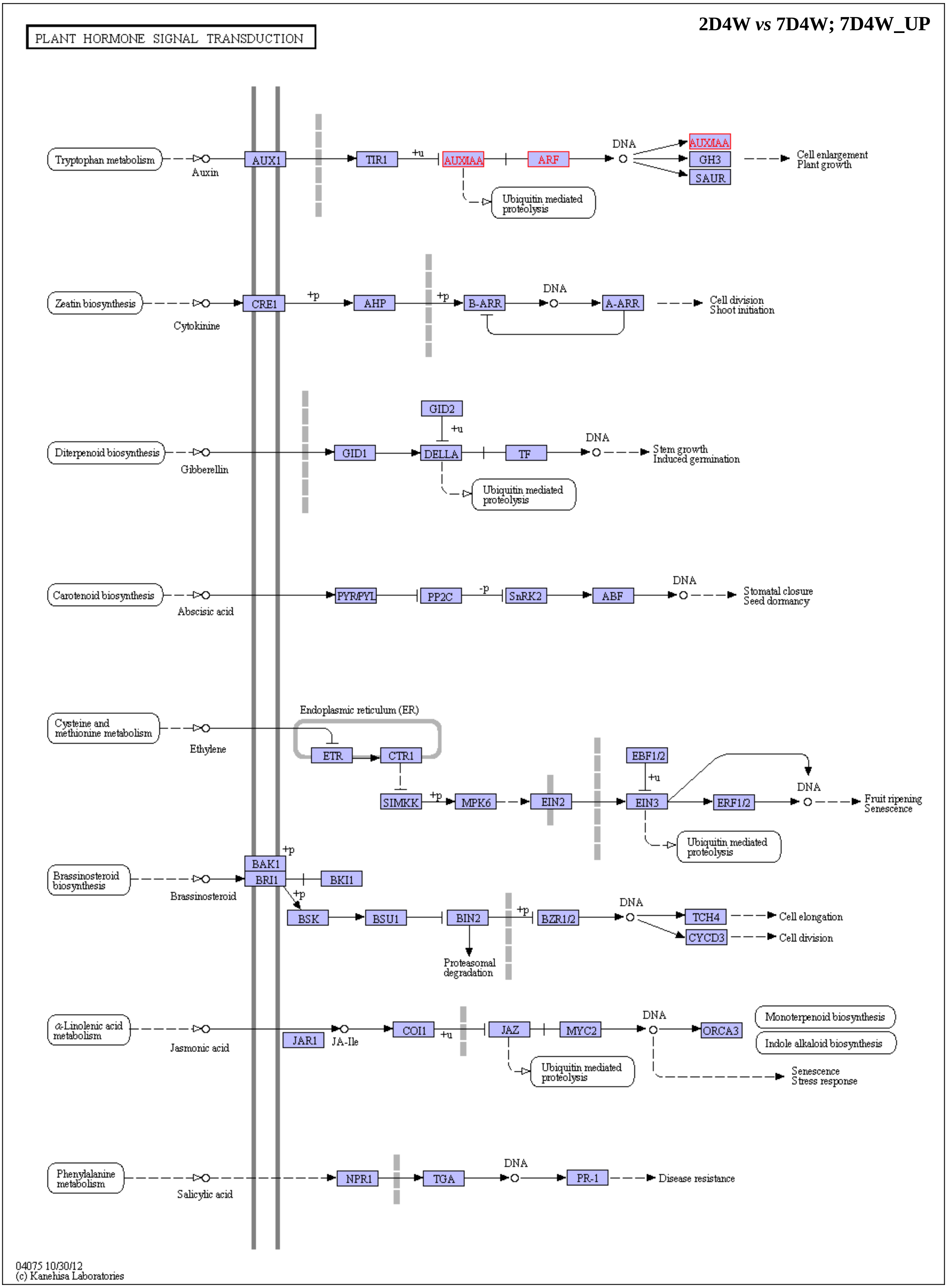

PLANT HORMONE SIGNAL TRANSDUCTION
2D4W vs 7D4W; 7D4W_UP
Tryptophan metabolism
Auxin
AUX1
TIR1
+u
AUX/IAA
ARF
DNA
AUX/IAA
GH3
SAUR
Cell enlargement
Plant growth
Ubiquitin mediated proteolysis
Zeatin biosynthesis
Cytokinine
CRE1
+p
AHP
+p
B-ARR
DNA
A-ARR
Cell division
Shoot initiation
GID2
+u
Diterpenoid biosynthesis
Gibberellin
GID1
DELLA
TF
DNA
Stem growth
Induced germination
Ubiquitin mediated proteolysis
Carotenoid biosynthesis
Abscisic acid
PYR/PYL
PP2C
-p
SnRK2
ABF
DNA
Stomatal closure
Seed dormancy
Endoplasmic reticulum (ER)
Cysteine and methionine metabolism
Ethylene
ETR
CTR1
EBF1/2
+u
DNA
SIMKK
+p
MPK6
EIN2
EIN3
ERF1/2
Fruit ripening
Senescence
Ubiquitin mediated proteolysis
+p
BAK1
Brassinosteroid biosynthesis
Brassinosteroid
BRI1
BKI1
+p
BSK
BSU1
BIN2
+p
BZR1/2
DNA
TCH4
CYCD3
Cell elongation
Cell division
Proteasomal degradation
α-Linolenic acid metabolism
Jasmonic acid
JAR1
JA-Ile
COI1
+u
JAZ
MYC2
DNA
ORCA3
Monoterpenoid biosynthesis
Indole alkaloid biosynthesis
Ubiquitin mediated proteolysis
Senescence
Stress response
Phenylalanine metabolism
Salicylic acid
NPR1
TGA
DNA
PR-1
Disease resistance
04075 10/30/12
(c) Kanehisa Laboratories
