# Supplementary material for: Transcriptome analysis of seed dormancy after rinsing and chilling in ornamental peaches (Prunus persica (L.) Batsch)
Source: BMC Genomics. 2016 Aug 8;17:575. doi: 10.1186/s12864-016-2973-y (PMC4977653; doi:10.1186/s12864-016-2973-y)
Supplement: Additional file 8: — Candidate-gene annotation by Swiss-Prot database. Candidate-gene involved in biosynthesis and catabolism of ABA, GA and stress response genes. (PDF 95 kb) [file 12864_2016_2973_MOESM8_ESM.pdf]

Trinity transcrip name	Annotation from the SwissProt database	BR *vs* 2D4W		BR *vs* 7D4W		2D4W *vs* 7D4W	
		**logFC**	**FDR**	**logFC**	**FDR**	**logFC**	**FDR**
**ABA biosynthesis**							
TR8162|c0_g1_i1	ABI five-binding protein 3 [*Arabidopsis thaliana*]	-0.910	7.04E-12	2.095	6.10E-22	-3.054	1.12E-40
TR8743|c0_g2_i1	9-cis-epoxycarotenoid dioxygenase NCED1 [*Phaseolus vulgaris*]	-6.157	1.69E-28	0.082	1.00	-6.307	3.71E-30
TR1393|c0_g1_i1	ABA-HYPERSENSITIVE GERMINATION 3; Protein phosphatase 2C A [*Arabidopsis thaliana*]	0.525	0.0310	3.136	1.16E-56	-2.664	6.35E-30
TR8214|c0_g1_i1	ABI five-binding protein 2 [*Arabidopsis thaliana*]	1.208	2.08E-15	3.405	3.06E-100	-2.239	2.57E-25
TR13511|c0_g2_i1	Abscisic acid 8'-hydroxylase 3; Cytochrome P450 707A3 [*Arabidopsis thaliana*]	-4.664	4.69E-05	-0.919	0.889	-3.911	0.0027
**GA biosynthesis**							
TR9163|c0_g2_i1	Gibberellin 2-beta-hydroxylase 8 [*Arabidopsis thaliana*]	-0.725	0.175	0.490	0.474	-1.274	0.0034
**MADS box**							
TR15408|c4_g2_i5	MADS-box protein JOINTLESS, LeMADS [*Solanum lycopersicum*]	3.056	4.23E-88	3.733	1.18E-152	-0.732	2.78E-02
**Stress respond**							
TR24454|c0_g1_i1	RecName: Full=EID1-like F-box protein 3 [*Arabidopsis thaliana*]	-3.474	1.58E-41	1.878	0.0005	-5.413	4.92E-71
TR10580|c0_g2_i1	RecName: Full=Dehydration-responsive element-binding protein 2C [*Arabidopsis thaliana*]	0.472	1.63E-05	3.826	5.90E-91	-3.411	1.10E-54
TR10071|c0_g2_i1	Late embryogenesis abundant protein D-34 [*Gossypium hirsutum*]	3.771	5.18E-109	5.979	3.55E-153	-2.237	0.0002
