# Supplementary material for: Transcriptome analysis of seed dormancy after rinsing and chilling in ornamental peaches (Prunus persica (L.) Batsch)
Source: BMC Genomics. 2016 Aug 8;17:575. doi: 10.1186/s12864-016-2973-y (PMC4977653; doi:10.1186/s12864-016-2973-y)
Supplement: Additional file 11: — Gene-specific primers for qRT-PCR. (PDF 182 kb) [file 12864_2016_2973_MOESM11_ESM.pdf]

Proteins	Forward primer (5’ to 3’)	Reverse primer (5’ to 3’)
**EID1-like F-box protein 3**	ATGTTCTTTTGCTGCGGCTG	GCTGACGTACAGCAAATCGC
**DREB2C**	GGCTATGTATGGTCCGGCTG	GGAGTTGCCACTGCAGAAGA
**ABI5-binding protein 3 (AFP3)**	ATTGCCGGAACTATGCCGTT	TCTGTCTCCGTGGGAAGTGA
**NCED1**	GCCAGTACCCCAGCAATGGA	CCGGCGATTTGGACTTTTGG
**PP2CA**	GGCTGCTCTCATGTGGCCTT	TCCTGGACCTCATCGTCCAT
**ABI5-binding protein 2 (AFP2)**	CATGAAGGTGGTGGAAGCAG	GGAGGTAGCTGTGTGTTCAG
**LEA D-34**	CCGAGAGGACATCTGTGAGC	CGGGCAGCAATGTGATAAGC
**ABA 8'-hydroxylase 3**	GGTTGGTGCACAAAATGGCA	GTGTCTGGAGTGTTATGGGCT
**GA2-oxidase 8**	CTTTCAGCCTTGTGCTAGAA	CGTACAGAGCCAGTAGAATG
**MADS-box protein JOINTLESS**	GCTGAGCTGGTAGAAGCCAA	GCCGTCTTCTTCTCCTCCAC
**UBQ**	CGAACCCTAGCCGATTACAA	AGTGGTTCGCCATGAAAGTC
